# Correlation between gut microbiota and their metabolites and the efficacy of chemotherapy combined with immunotherapy for extensive-stage small cell lung cancer

**DOI:** 10.3389/fonc.2025.1683347

**Published:** 2026-03-03

**Authors:** Wenjing Song, Shiwei Liu, Dan Zang, Wenjuan Meng, Chenguang Liu, Jun Chen

**Affiliations:** 1 Medical Oncology, The Second Hospital of Dalian Medical University, Dalian, Liaoning, China; 2 Oncology Department, Weifang People’s Hospital, Shandong Second Medical University, Weifang, Shandong, China; 3 Joint Surgery Department, Weifang People’s Hospital, Shandong Second Medical University, Weifang, Shandong, China

**Keywords:** biomarker, efficacy, gut microbiota and metabolites, immunotherapy, small cell lung cancer

## Abstract

**Introduction:**

Gut microbiota has been reported to be associated with the host’s immune system and immunotherapy response, as well as immune-related adverse events (irAEs). Additionally, gut microbial metabolites have various immunomodulatory effects. Our study focused on the differences in gut microbiota and their metabolites between long progression-free survival (PFS) and short PFS in patients with small cell lung cancer before and after chemotherapy combined with immunotherapy.

**Methods:**

The enrolled patients collected in our department were divided into long PFS and short PFS groups according to whether the PFS was ≥6 months, and the stool samples before and after treatment were analyzed using metagenomics and metabolomics.

**Results:**

The results showed that *Streptococcus* (P = 0.00648), *Actinomyces* (P = 0.0124), and *Roseburia* (P = 0.0127) differed between the long and short PFS groups. In the analysis of differential metabolites, we found that indirubin-3’-monoxime (AUC 0.611), stearidonic acid (AUC 0.867), leukotriene B4 (AUC 0.844), trans-cinnamic acid (AUC 0.792), and L-tyrosine (AUC 0.751) could be used as potential biomarkers.

**Discussion:**

Gut microbiota and their metabolites hold broad prospects for translational applications in cancer clinical management, such as the development of microbial biomarkers and the modulation of microbiota to enhance the efficacy of chemotherapy and immunotherapy.

## 1 Introduction

Lung cancer (LC) is one of the most common and leading causes of cancer-related death worldwide (1, 2). It is estimated that in 2020, more than 2.2 million new cases of LC were diagnosed and more than 1.8 million people died of LC globally (3, 4). SCLC is characterized by an extremely high proliferation rate, strong tendency for early extensive metastasis, and acquired chemotherapy resistance. The prognosis of patients with SCLC is very poor, with a 5-year survival rate of less than 5%.The average overall survival (OS) of patients who do not receive any active treatment is only 2–4 months (5, 6). With a better understanding of immune checkpoints in tumor control, immunotherapy has revolutionized our treatment of various types of cancer, including SCLC (7, 8). In the IMpower133 study, atezolizumab combined with chemotherapy showed good efficacy as first-line treatment. This is the first phase III study of more than 30 years to achieve OS improvement in ES-SCLC. Although immunotherapy has become a major component of SCLC treatment, many challenges remain (9). Not all patients can benefit from immunotherapy, and some patients even develop immune-related adverse events (irAEs) that cannot be ignored, such as potentially fatal pneumonia or myocarditis. The identification of predictive biomarkers to select patients who are most likely to benefit from this treatment is an area of significant unmet need (10).

In recent years, the relationship between gut microbiota and human health and disease has been increasingly recognized. A large number of studies have shown that gut microbiota is closely related to the occurrence and development of various diseases, including tumors, neurological, cardiovascular, digestive, and other diseases, and their causal relationships are also gradually being clarified (11–13). Several studies have shown that the gut microbiota is associated with the host immune system, immunotherapy response, and irAEs (14–17). For example, *Akkermansia muciniphila* was enriched in NSCLC patients who responded to programmed death-1 (PD-1)-based immunotherapy (18). The relative abundance of *Akkermansia muciniphila* was also found to be a clinical predictor of poor response to immune checkpoint inhibitors in renal cell carcinoma patients (19). In the clinical setting, enrichment of *Faecalibacterium* and other genera of the *Firmicutes* phylum was also associated with a favorable clinical response to ICIs (20). Another study found that the efficacy of anti-PD-L1 therapy was associated with the enrichment of *Enterococcus faecium, Collinsella aerofaciens*, and *Bifidobacterium longum* (21). In LC patients receiving immunotherapy, enrichment of *Bifidobacterium* was associated with a lower incidence of irAEs (22). 16S rRNA gene sequencing analysis showed that *Bifidobacteria* are involved in tumor control. Bifidobacteria can stimulate the function of dendritic cells (DCs) in tumors and increase the infiltration of CD8+ T cells, thereby enhancing the efficacy of Programmed Death-Ligand 1 (PD-L1) and controlling tumor progression (23). Not only that, microbial metabolites such asshort-chain fatty acids (SCFAs), inosine, and bile acids have various immunomodulatory functions (24). Microbiota-derived SCFAs, particularly butyrate, enhance the effector functions of CD8+ T cells by remodeling cellular metabolism and boosting mitochondrial respiration and oxidative phosphorylation (25). Compared with responders to anti-PD-1 immunotherapy, non-responders have a lower abundance of bacteria that produce SCFAs, such as Akkermansia muciniphila and Faecalibacterium (18, 20, 26). Butyric acid, an SCFA, can increase the levels of retinoic acid and IL-10 in the intestinal microenvironment, thereby promoting the proliferation and differentiation of regulatory T cells (27). CD8+ T cells are regarded as the core executors of antitumor immunity (28). Butyric acid increases histone 3 lysine 27 acetylation at promoter regions in human CD8+ T cells, thereby promoting the expression of PD-1/CD28 and enhancing the efficacy of anti-PD-1 therapy (29). Butyric acid supplementation facilitates the expression of anti-tumor cytokines in cytotoxic CD8+ T cells by modulating the T cell receptor signaling pathway (29). Butyrate can suppress histone deacetylase (HDAC), thereby upregulating the transcriptional regulators IL-12R and ID2 signaling pathways in CD8+ T cells, and enabling CD8+ T cells to activate and expand (30). *In vitro* studies have confirmed that butyrate and valerate treatment of CTLs and CAR T cells can activate mTOR, a central cellular metabolic sensor, and inhibit the activity of class I HDAC (31). This metabolic reprogramming enhanced the secretion of effector molecules such as IFN-γ, CD25, and TNF-α. Consequently, it significantly improved the antitumor efficacy of both ROR1-targeting CAR-T cells and antigen-specific CTLs in syngeneic mouse models of melanoma and pancreatic cancer. Butyrate is expected to become a powerful ally in cancer treatment. In human cancer patients, the level of butyrate is positively correlated with the efficacy of oxaliplatin. Butyrate can enhance the therapeutic effect of anti-PD-L1 treatment, but it is ineffective for non-immunogenic drugs such as cisplatin, possibly because its efficacy is less dependent on immune responses (26). In patients with cirrhotic portal vein thrombosis, the gut microbiota is severely disrupted, primarily characterized by an increase in Gram-negative pathogenic bacteria and a decrease in beneficial bacteria that produce SCFAs (32). A study by Popescu et al. found that *Bacteroides* exerts anti-inflammatory effects by producing SCFAs, which may indirectly reduce the risk of thrombosis (33). Research has shown that butyrate can regulate the inflammatory response and oxidative stress in endothelial cells by inhibiting HDAC, processes that are crucial in thrombosis formation (32). Inosine, a purine metabolite derived from *Bifidobacterium pseudolongum*, can bind to the adenosine 2A receptor (A2AR) on T cells and activate the initiation of the inosine-A2AR-cAMP-PKA signaling pathway, which induces the differentiation of naive T cells into CD4+ Th1 cells in response to the co-stimulation of DCs (34). The combination of inosine and PD-L1 inhibitor can increase the secretion of IFN-γ and infiltration of CD8+T cells compared with the PD-L1 inhibitor alone (35). Beyond addition, inosine is an alternative energy source for glucose in cytotoxic T lymphocytes (CTL); however, tumor cells cannot use inosine. Hence, inosine supplementation can accelerate the damage of tumor cells by CTL (36).

A study using gut microbiota to predict the efficacy of immunotherapy in NSCLC observed that Kyoto Encyclopedia of Genes and Genomes (KEGG) Orthology, Pfam, and combined profiles ranged from 97% to 100% of AUC and 98.4% to 99.9% of F1 score, achieving a near-perfect prediction (37). To date, there has been no published prediction model using gut microbiota to predict the efficacy of immunotherapy in SCLC. Therefore, our study utilized metagenomics and metabolomics to investigate the differences in gut microbiota and metabolites between patients with long and short progression-free survival (PFS) of ES-SCLC treated with chemotherapy combined with immunotherapy. We aimed to establish a prognostic prediction model and determine the biological processes underlying the differences between long and short PFS in ES-SCLC as much as possible.

## 2 Research object and methods

### 2.1 Research object

This study was approved by the Ethics Committee of the Second Affiliated Hospital of the Dalian Medical University (Ethics Statement Number:2023-186). Baseline fecal samples before anti-tumor treatment and fecal samples at the progression of first-line chemotherapy combined with immunotherapy were collected from SCLC patients admitted to the Department of Thoracic Oncology 1, Department of Medical Oncology, Second Affiliated Hospital of Dalian Medical University from May 2021 to October 2023.

The inclusion criteria were as follows: 1) cytological or pathological diagnosis of SCLC; 2) KPS≥60, ECOG ≤ 2; 3) patients who had never received treatment, including surgery, chemotherapy, immunotherapy, targeted therapy, and radiotherapy; exclusion criteria: 1)other acute and chronic diseases that affect the composition of the gut microbiota, such as mental illnesses, metabolic diseases, and other cancers; 2) receiving antibiotic treatment within the past 3 months; and 3) pregnancy or lactation.

### 2.2 Enrollment status

**Figure 1:**
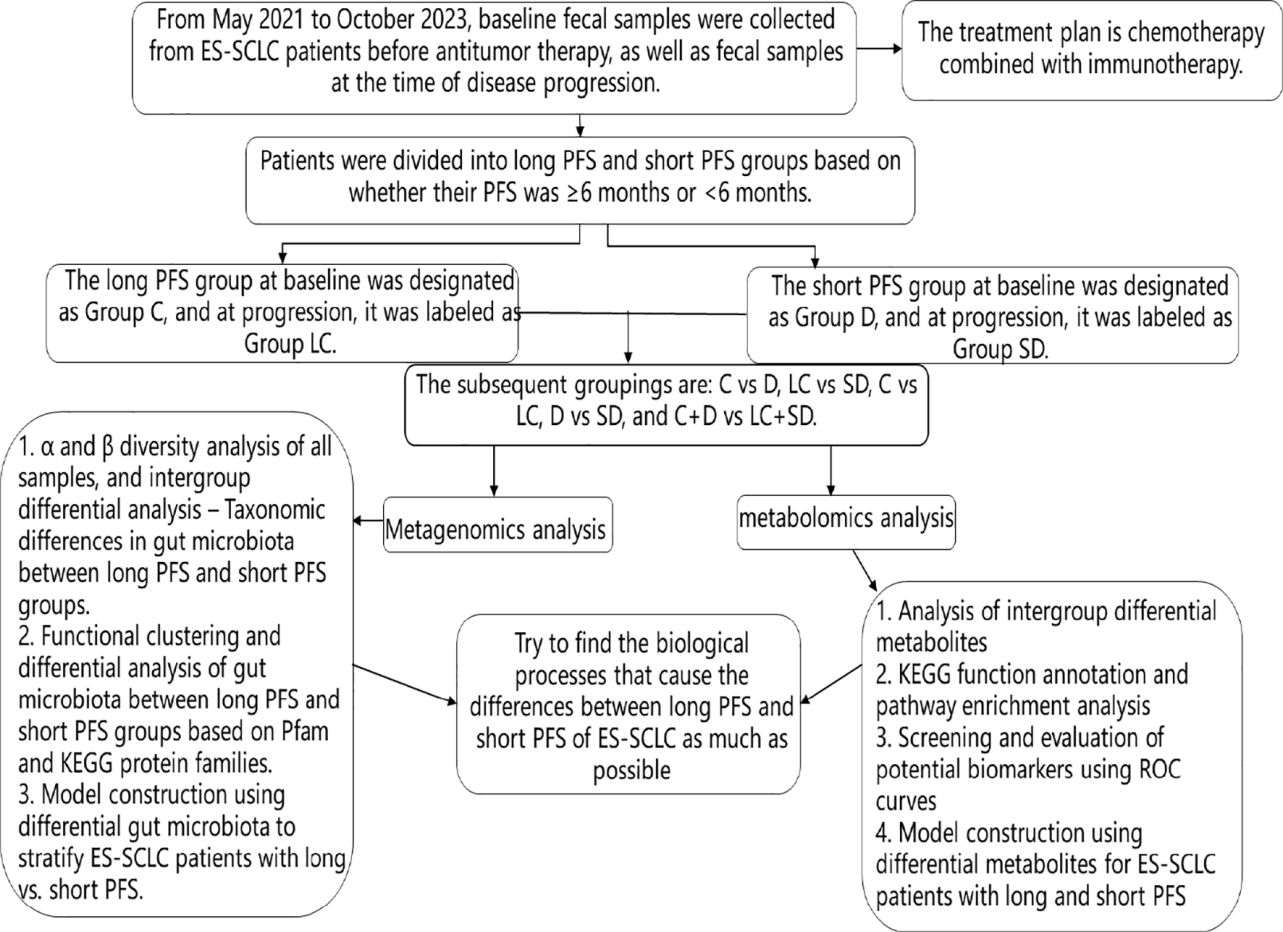
Technical roadmap.

A total of 105 patients with SCLC were screened from May 2021 to October 2023;79 patients with no baseline fecal samples, patients with treatment options other than chemotherapy combined with immunotherapy, patients with unknown PFS, and untreated patients were removed, and 26 patients were finally included. PFS≥6 months was divided into the long PFS group; PFS < 6 months was the short PFS group, in which 18 patients with long PFS were in group C and 8 patients with short PFS were in group D. Fecal samples from these 26 patients were collected again when the patients progressed on first-line chemotherapy combined with immunotherapy. Among them, 5 patients did not retain fecal samples when they progressed, and 21 patients were finally enrolled. At disease progression, 15 patients in the long PFS group were classified as the LC group, and 6 patients in the short PFS group were classified as the SD group. The information of the enrolled patients is presented in **Table 1**.Subsequently, we conducted a comparative analysis at five levels: 1. Before anti-tumor treatment, we will analyze the C *vs*. D group at baseline to reveal the differences in gut microbiota and metabolites of patients with long and short PFS. 2. When chemotherapy combined with immunotherapy progresses, LC *vs*. SD will be analyzed to reveal the changes in gut microbiota and metabolites of patients with long and short PFS after anti-tumor therapy compared with baseline. 3. When anti-tumor therapy progresses, we will analyze C *vs*. LC to reveal the effects of anti-tumor therapy on gut microbiota and metabolites in patients with long PFS. 4. When anti-tumor therapy progresses, D *vs*. SD will be analyzed to reveal the effects of anti-tumor therapy on gut microbiota and metabolites in patients with short PFS. 5. Finally, we will analyze the changes in gut microbiota and metabolites after anti-tumor treatment in the whole population (i.e., C+D *vs*. LC+SD) to reveal more about the influence of chemotherapy combined with immunotherapy on gut microbiota changes. The technology roadmap is shown in **Figure 1**.

**Table 1:** Basic information of enrolled patients.

Long PFS group	Average age	Sex	Number of people	Ethnicity	Staging	Treatment	Number of cases	PFS
	70.56	Male	17	all are of the Han Chinese	all were in the extensive stage	EC+Durvalumab	4	8.27
		Female	1	the Han Chinese	extensive stage	EP+Durvalumab	8	7.79
						EP+Serplulimab	1	8.80
						EC+Serplulimab	2	11.03
						EC+Atezolizumab	1	7.70
						EP+Atezolizumab	1	6.10
						EC+Tislelizumab	1	6.03
								
Short PFS group	Average age	Sex	Number of people	Ethnicity	Staging	Treatment		PFS
	67.73	Male	6	all are of the Han Chinese	all were in the extensive stage	EC+Durvalumab	1	3.70
		Female	2	all are of the Han Chinese	all were in the extensive stage	EP+Durvalumab	1	3.97
						EP+Serplulimab	1	1.40
						EC+Serplulimab	1	0.77
						EC+Atezolizumab	1	2.07
						EP+Atezolizumab	1	1.57
						E+Durvalumab	2	1.17

### 2.3 Research methods

The collected feces were subjected to metagenomic and metabolomic analyses. The specific process is as follows.

#### 2.3.1 Experimental scheme

##### 2.3.1.1 Metagenomic experiment process

The experimental procedures were performed according to the standard protocol provided by Illumina, including sample quality assessment, library preparation, library quality control and sequencing. DNA passing quality assessment was fragmented. DNA fragments were further processed for end repairing, adding 3’ A tail and adapter ligation. The products of proper size were selected and purified. The selected fragments were further amplified to construct sequencing library. Qualified libraries were then processed for sequencing on Illumina platform.

##### 2.3.1.2 Metabolomic experiment process

The metabolite detection process includes metabolite extraction, on-machine detection, and the qualitative quantification of metabolites.

###### 2.3.1.2.1 Metabolite extraction

The main steps of metabolite extraction include adding an appropriate amount of extraction solution and magnetic beads for grinding and ultrasonic treatment, centrifuging and then taking the supernatant for vacuum drying, and then adding an appropriate amount of extraction solution for reconstitution and testing on the machine.

###### 2.3.1.2.2 On-machine detection

The detection platform was a Waters Acquity I-Class PLUS ultra-performance liquid chromatography tandem Waters Xevo G2-XS QTOF high-resolution mass spectrometer, and sample detection was analyzed according to the corresponding parameters.

###### 2.3.1.2.3 Qualitative quantification of metabolite

The raw data collected by MassLynx V4.2 were processed through the Progenesis QI software for peak extraction, peak alignment and other data processing operations. The Progenesis QI software is used for identification based on the online METLIN database, public databases and Baimak’s self-built database, and at the same time, theoretical fragment identification is performed. The precursor ion mass number deviation was within 100ppm, and the fragment ion mass number deviation was within 50ppm.

#### 2.3.2 Bioinformatics workflow

##### 2.3.2.1 Metagenomic bioinformatics workflow

Raw data quality control was performed to clean reads for subsequent bioinformatics analyses. Clean reads were firstly assembled to predict coding genes and construct a non-redundant gene set. Function annotation, taxonomy analysis, and statistics on abundance were further performed on base of this gene set according to existing databases.

##### 2.3.2.2 Metabolomic bioinformatics workflow

After qualitative quantification of metabolites, data quality assessment, annotation analysis, differential expression analysis, and functional enrichment were performed. Metabolomics data possess the characteristics of being “high-dimensional” and “massive.” Therefore, it is necessary to combine univariate statistical analysis and multivariate statistical analysis methods and analyze the data from multiple perspectives in order to accurately identify differential metabolites. Univariate statistical analysis methods include parametric tests and non-parametric tests. Multivariate statistical analysis methods include Principal Component Analysis (PCA) and Orthogonal Partial Least Squares Discriminant Analysis (OPLS-DA). Based on the results of OPLS-DA, differential metabolites can be preliminarily selected by analyzing the Variable Importance in Projection (VIP) obtained from the multivariate analysis of the OPLS-DA model. Additionally, the p-value from univariate analysis or the fold change value can be used to further filter the differential metabolites. The selection criteria are as follows:

1. Select metabolites with a fold change ≥ 1. If the difference in metabolite abundance between the control group and the experimental group is above it is considered as significant difference.
2. In addition to the above criteria, select metabolites with a VIP value ≥ 1. The VIP value indicates the impact strength of the between-group difference of the corresponding metabolite in the sample classification and discrimination of the model. Generally, metabolites with VIP ≥ 1 are considered significantly different.
3. Further filter differential metabolites by selecting those with a p-value <0.05 using the t test. Generally, metabolites with a p-value < 0.05 are considered significantly different.

We have uploaded the raw data of metagenomics and metabolomics sequencing results to NCBI and MetaboLights with the accession number PRJNA1391873 and MTBLS12382.

## 3 Results

### 3.1 Metagenomic and metabolomic results of C *vs* D

First, we conducted a comparative analysis of metagenomics and metabolomics on stool samples from patients with long and short PFS before anti-tumor treatment.

#### 3.1.1 Metagenomic results

##### 3.1.1.1 Gene number difference and function annotation

By analyzing the difference in gene numbers between groups, it was concluded that there was no significant difference in the number of genes between the two groups.

Among the cellular components annotated by gene ontology (GO) function, it was mainly concentrated in the cell membrane, followed by the cell. In terms of molecular function, it mainly focuses on catalytic activity and binding. In terms of biological processes, it mainly focuses on metabolic processes and cellular processes, and it can also be seen that it plays a role in biological regulation (such as neuromodulation and endocrine regulation) and localization.

In the KEGG functional annotation, in terms of metabolism, it mainly focuses on carbohydrate metabolism, amino acid metabolism, nucleotide metabolism, cofactors and vitamin metabolism; in terms of genetic information processing, it mainly focuses on translation, replication and repair; in terms of environmental information processing, it mainly focuses on membrane transport and signal transduction.

##### 3.1.1.2 Beta diversity analysis of functional genes

From the PCA plot (**Supplementary Figure 1A**) and PCoA plot (**Supplementary Figure 1B**) of functional genes, it can be seen that there is no significant difference in the Beta diversity of functional genes between the two groups.

##### 3.1.1.3 Differential analysis of functional genes

**Figure 2:**
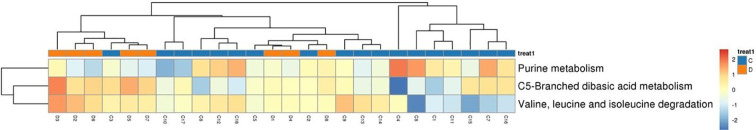
Abundance heat map on differential functional genes in C vs D group. Functional genes with p-value smaller than 0.05 in parametric differential test were shown in the heat map. Each row stands for a gene and each column stands for a sample. The first line shows grouping information. The color indicates normalized relative abundance.

Functional gene differences between groups were reflected in purine metabolism, C5-branched dibasic acid metabolism, and degradation of valine, leucine, and isoleucine (**Figure 2**).

##### 3.1.1.4 Taxonomy diversity

###### 3.1.1.4.1 Histogram of taxonomy composition

The species composition distribution map (**Supplementary Figure 2**) shows the species histogram at the family level. From the figure, the species composition of each sample and the proportion of different species in each sample can be seen. It can be seen that the bacteria in the feces of the patients are mainly *Enterobacteriaceae, Oscillospiraceae, Bacteroidaceae, Lachnospiraceae, Prevotellaceae, Clostridiaceae, Eubacteriaceae, Veillonellaceae, Rikenellaceae, Selenomonadaceae, Tannerellaceae, Chitinophagaceae, Acidaminococcaceae, Streptococcaceae, Bifidobacteriaceae*, etc.

###### 3.1.1.4.2 Alpha diversity analysis and beta diversity analysis of taxonomy

**Figure 3:**
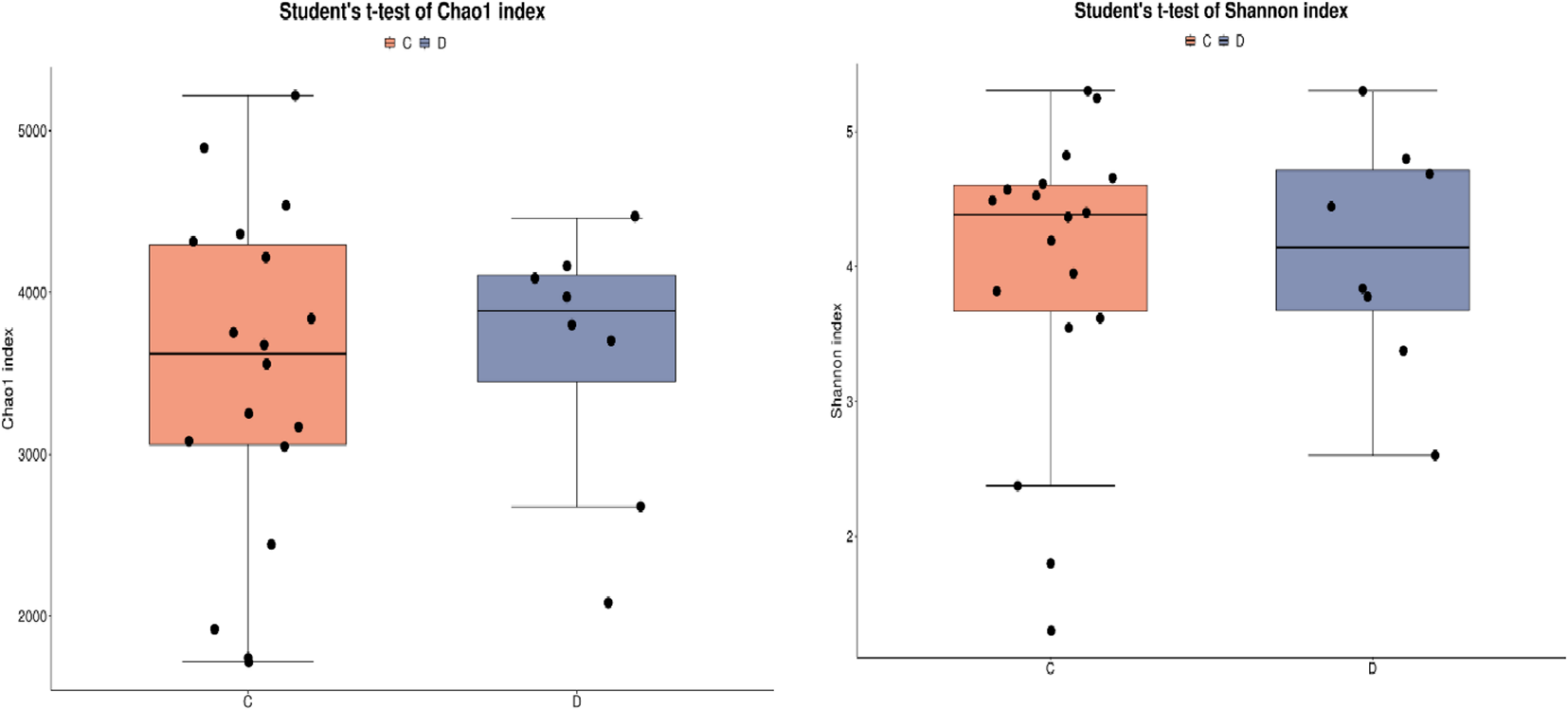
**(A)** Chaol index in C vs D group **(B)** Shannon index in C vs D group.

Alpha diversity reflects the taxonomy richness and diversity of individual samples. The Chao1 index measures taxonomy richness, that is, the number of species. The Shannon index is used to measure taxonomy diversity and is affected by taxonomy richness and community evenness. In the case of the same taxonomy richness, the greater the evenness of each taxonomy in the community, the greater the diversity of the community. The greater the Shannon index value, the higher the taxonomy diversity of the sample. There was no significant difference in Chao 1 index (**Figure 3A**) and Shannon index (**Figure 3B**) between the two groups. From the PCA plot and PCoA plot of species abundance, it can be seen that there was no significant difference in the beta diversity of species between the two groups.

##### 3.1.1.5 Differential analysis of taxonomy

**Figure 4:**
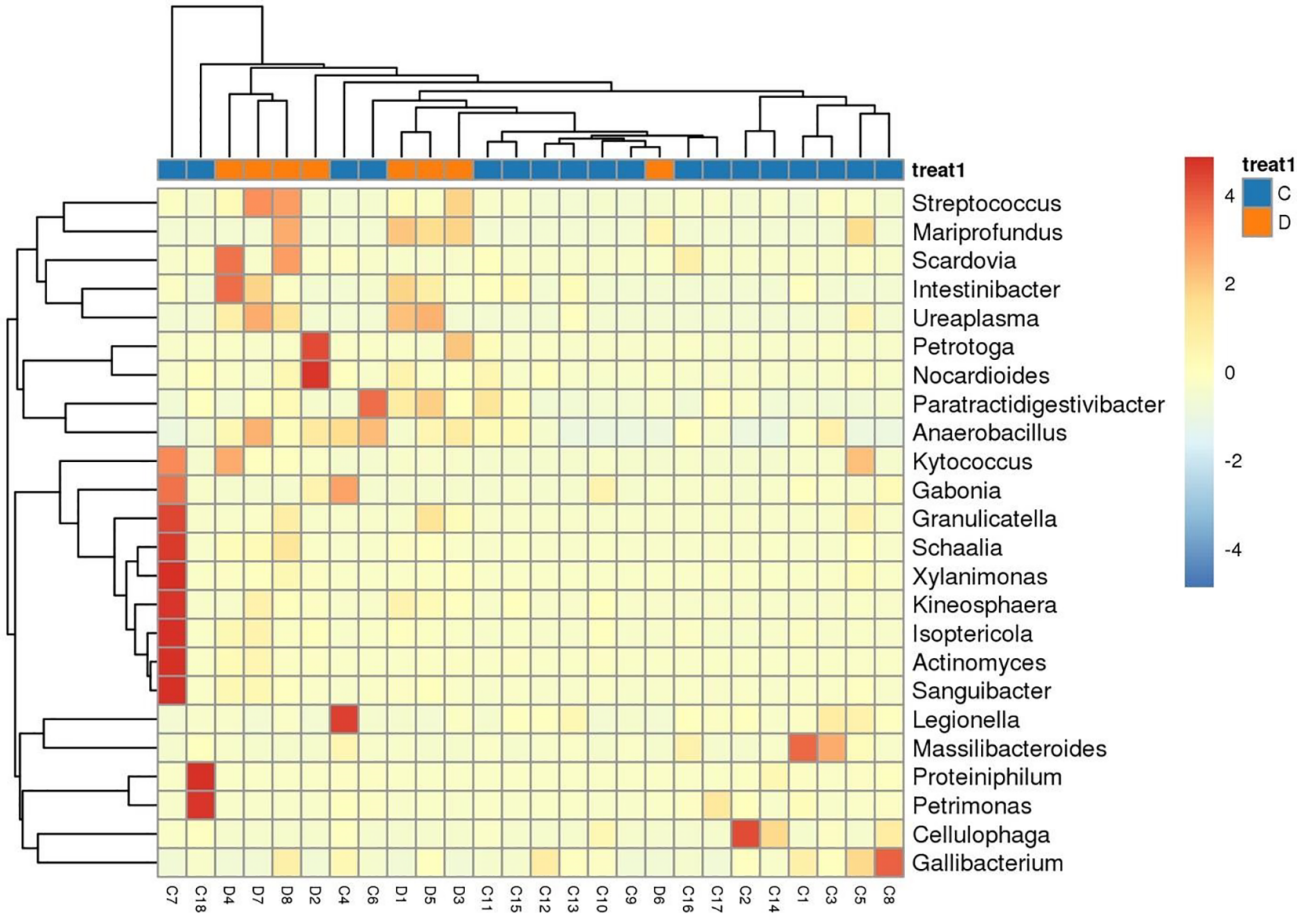
Genus-level heat map of differential species abundance in C vs D group. The species shown in the figure are species with a p-value less than 0.05 after rank sum test. The cluster tree on the left is the differential species cluster tree, the cluster tree on the top is the sample cluster tree, and the middle is the heat map.

**Figure 5:**
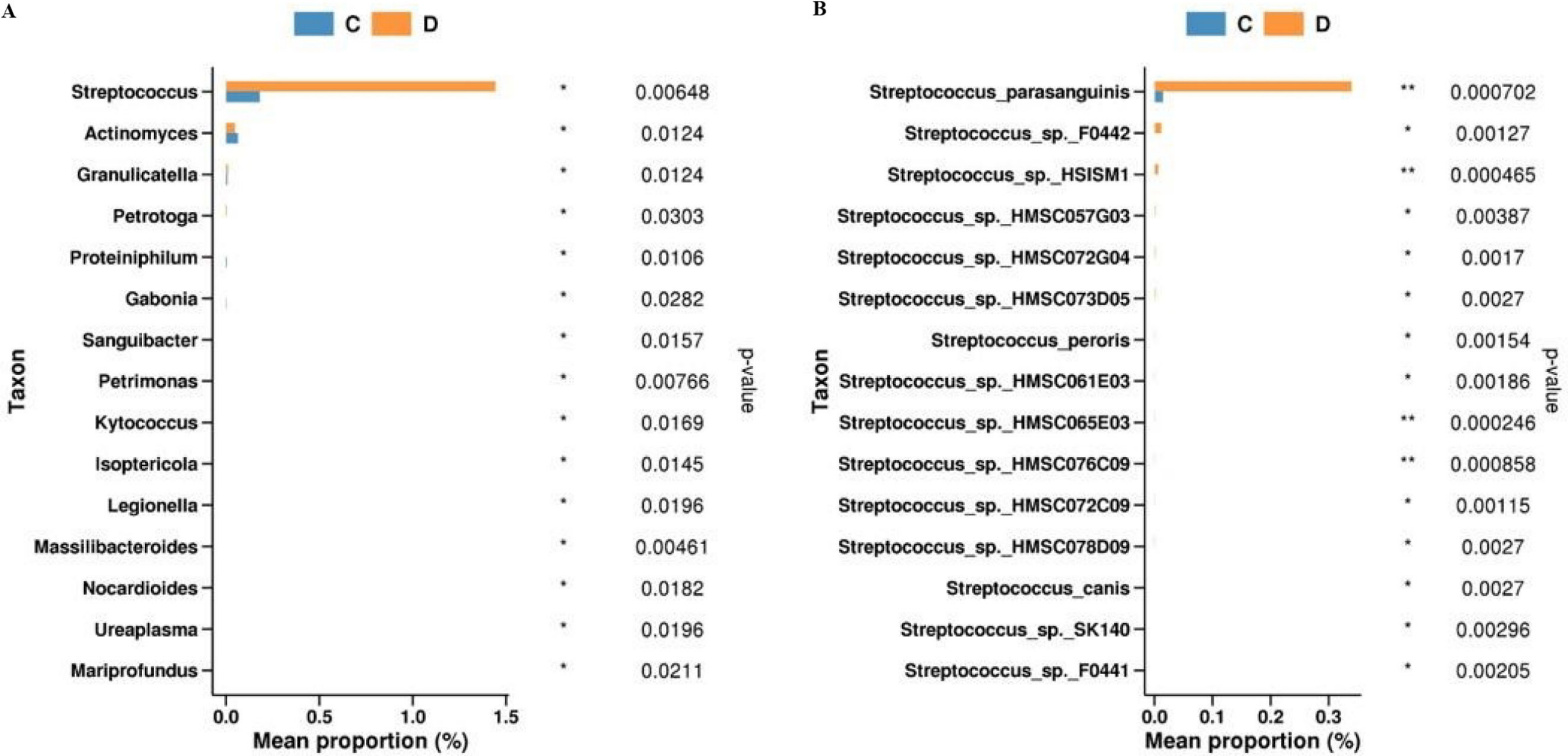
**(A)** Histogram of differential species abundance by genus level rank sum test in C vs D group **(B)** Histogram of differential species abundance by species level rank sum test in C vs D group.

Analysis of differential species between groups (**Figure 4**) revealed that *Streptococcus, Actinomyces, Granulicatella, Intestinibacter, Petrotoga, Isoptericola, Anaerobacillus, Petrimonas* showed differences in baseline samples of patients with long and short PFS, among which *Streptococcus* (P = 0.00648) was highly expressed in the short PFS group, and *Actinomyces* (P = 0.0124) was highly expressed in the long PFS group (**Figure 5A**). From the species-level differential species abundance histogram (**Figure 5B**), it can be seen that the differential species were all *Streptococcus bacteria*, with *Streptococcus parasanguinis* accounting for the majority.

##### 3.1.1.6 Random forest analysis of taxonomy

The species importance ranking diagram constructed by random forest analysis is shown in **Supplementary Figure 3**. *Megasphaera, Actinomyces, Streptococcus, Butyricicoccus, Intestinibacter, Granulicatella, Petrimonas, Sanguibacter, Serratia* were highly important for sample classification.

#### 3.1.2 Metabolomic results

##### 3.1.2.1 PCA

It can be seen from the PCA diagram (**Supplementary Figure 4**) that the confidence circles of fecal metabolites in patients with long and short PFS are not separated at baseline. Later, we compared the changes in the confidence circles of metabolites in LC *vs*. SD, C *vs*. LC, D *vs*. SD, and C+D *vs*. LC+SD after treatment.

##### 3.1.2.2 KEGG database annotation

From the KEGG database annotation of metabolites (**Supplementary Figure 5**), it can be seen that metabolites are mainly concentrated in neomycin, kanamycin, gentamicin biosynthesis, steroid hormone biosynthesis, bile secretion, arachidonic acid metabolism, ABC transporter, insect hormone biosynthesis, porphyrin metabolism, drug metabolism - cytochrome P450, tryptophan metabolism, etc.

##### 3.1.2.3 Differential metabolite analysis

The results are presented in **Table 2**.

**Table 2:** Statistical of differential metabolites.

Group	Total_num	Diff_num	Up_num	Down_num
**D_*vs*_C**	991	7	6	1

Total_num: The total number of metabolites in Group C and Group D.

Diff_num: The number of differential metabolites between Group C and Group D.

Up_num: The number of metabolites upregulated in Group C compared to Group D.

Down_num: The number of metabolites downregulated in Group C compared to Group D.

###### 3.1.2.3.1 Difference fold analysis

**Figure 6:**
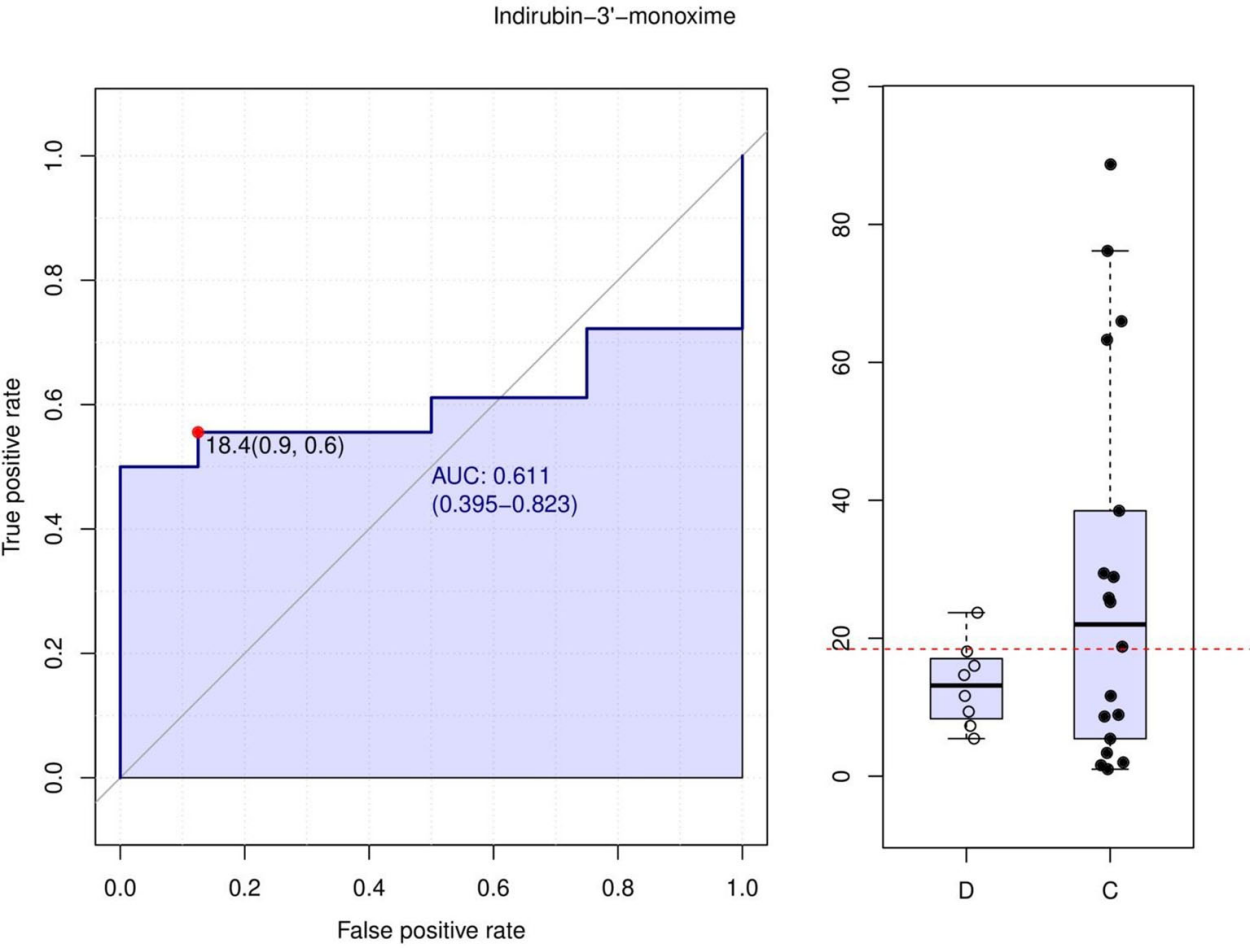
Indirubin-3’-monoxime.

After qualitative and quantitative analyses of the detected metabolites, the Fold Change (FC) of the quantitative information of metabolites in each group was compared. **Supplementary Figure 6** shows the logFC results of the upregulated and downregulated metabolites in group C compared to group D after log conversion for different multiples of different metabolites. In the differential metabolite analysis, a total of 991 metabolites were detected, of which seven were differential metabolites, six were upregulated, and one was downregulated. Among the differential metabolites detected, indirubin-3’-monoxime is a potent inhibitor of glycogen synthase kinase 3β (GSK-3β). Because GSK-3β phosphorylates tau protein, Indirubin-3’-monoxime can prevent tau phosphorylation-related sites in Alzheimer’s disease both *in vitro* and *in vivo*. It inhibits cyclin-dependent kinases at higher concentrations, reversibly inhibits the proliferation of multiple cell types, and prevents the G2/M phase cycle. Receiver operating characteristic (ROC) curve analysis method is commonly used to screen and evaluate biomarkers (38). Area under the curve (AUC) is a useful metric for measuring the ROC curve. Indirubin-3’-monoxime was higher in the long PFS group than in the short PFS group, and its AUC was 0.611 in the ROC curve analysis (**Figure 6**), which can be used as a potential biomarker.

#### 3.1.3 Metagenomic and metabolomic joint analysis

##### 3.1.3.1 Correlation analysis of differential metabolites and taxonomy abundance

We performed Pearson’s correlation analysis between differential metabolites and the abundance of differential species, and visualized the results in a heatmap (**Supplementary Figure 7**). Unfortunately, in the analysis of the correlation between differential metabolites and species abundance, we found *Streptococcus*, but not *Actinomyces*, and no correlation between them and the metabolite indirubin-3’-monoxime was found.

##### 3.1.3.2 Random forest classification analysis of metabolomic and metagenomic taxonomy abundance and metagenomic functional genes

**Figure 7:**
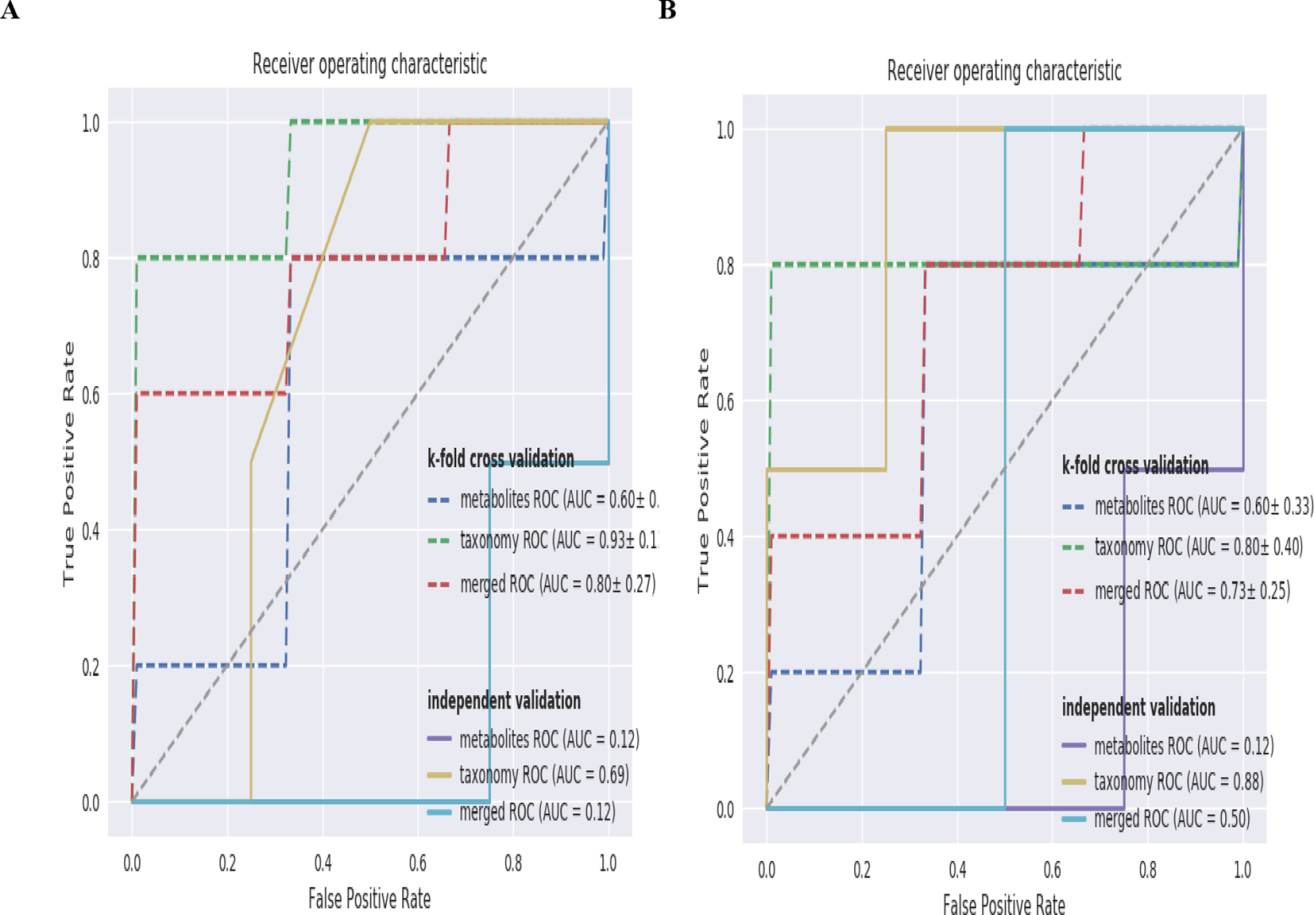
**(A)** ROC curve of metabolomic and metagenomic species abundance random forest classifier in C vs D group **(B)** ROC curve of metabolomic and metagenomic functional gene random forest classifier in C vs D group. **(A)** The dashed line shows the average ROC curves for the training set re-cross validation and the solid line shows the ROC curves for the independent validation. Different colored dashed and solid lines indicate the ROC curves for metabolomic and metagenomic taxonomy abundance modeled separately as well as combined modeling, respectively. **(B)** The dashed line shows the average ROC curves for the training set re-cross validation and the solid line shows the ROC curves for the independent validation. Different colored dashed and solid lines indicate the ROC curves for metabolomic and metagenomic functional gene modeled separately as well as combined modeling, respectively.

Random forest modeling is widely used in the modeling of data sets with few samples and high feature dimensions. In the combined analysis of multiple omics, the important features of the model can be selected by sequencing the model features, so as to screen the purpose of biomarkers. It is also possible to assess which omics better separates the control and experimental groups by comparing the ROC curves of different omics modeled individually with those modeled from the combined data. According to the ROC curves of metabolomic and metagenomic taxonomy abundance modeling separately and combined modeling (**Figure 7A**), we can see that the result of k-fold cross-validation modeling based on taxonomy abundance is satisfactory, with an AUC of 0.93 ± 0.13. In k-fold cross-validation modeling of metabolites, the AUC was 0.60 ± 0.33, showing poor results. Even though the combined modeling of metabolites and species abundance has an AUC of 0.80 ± 0.27, we believe that the modeling effect is not as good as that of species abundance alone. The AUC of k-fold cross validation modeling based on functional genes of gut microbiota was 0.80 ± 0.40 (**Figure 7B**), which was as good as when modeling via species abundance. The addition of metabolites to the combined model would only reduce the modeling effect.

### 3.2 Metagenomic and metabolomic results of LC *vs* SD

When the patient’s treatment progressed, we analyzed the gut microbiota again to reveal changes in the gut microbiota.

#### 3.2.1 Metagenomic results

##### 3.2.1.1 Basic situation

In LC *vs*. SD, GO functional annotation and KEGG functional annotation were the same as in the C *vs*. D group. There were no significant differences in the analysis of gene number differences, beta diversity analysis of functional genes, alpha diversity analysis of taxonomy, and beta diversity analysis of taxonomy between the LC *vs*. SD groups, as in the C *vs*. D group.

##### 3.2.1.2 Differential analysis of functional genes

After treatment, functional gene differences between groups were reflected in carbon metabolism, thiamine metabolism, biotin metabolism, and styrene degradation (**Supplementary Figure 8**).

##### 3.2.1.3 Differential analysis of taxonomy

**Figure 8:**
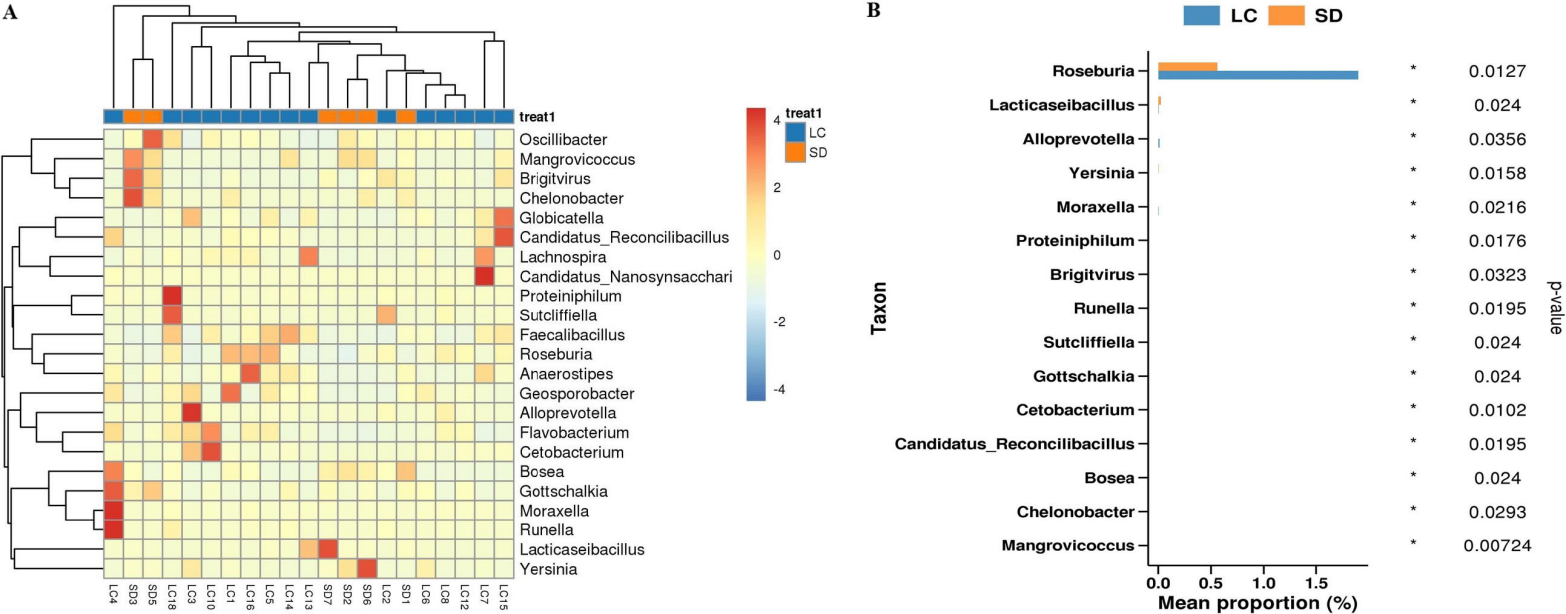
**(A)** Genus-level heat map on different species abundance in LC vs SD group **(B)** Histogram of differential species abundance by genus level rank sum test in LC vs SD group.

After treatment, the different species in the LC *vs*. SD group were mainly concentrated in *Roseburia, Lacticaseibacillus, Candidatus, Lachnospira, Yersinia, Faecalibacillus* (**Figure 8A**). Among them, *Roseburia* (P = 0.0127) was highly expressed in the long PFS group (**Figure 8B**), and at the species level, different species were reflected in the bacteria of *Eubacterium, Clostridiales, Candidatus and Roseburia*.

##### 3.2.1.4 Random forest analysis of taxonomy

From the random forest analysis diagram of species, it can be concluded that the importance of *Selenomonas, Roseburia, Oscillibacter, Alloprevotella, Desulfitobacterium, Lacticaseibacillus, Enterococcus, Barnesiella wa*s higher.

#### 3.2.2 Metabolomic results

##### 3.2.2.1 PCA

Compared with the PCA analysis of the C *vs* D group at the treatment baseline, the confidence circles of LC and SD in the PCA analysis of the LC *vs* SD group have a clear separation trend compared with the confidence circles of C and D at the baseline.

##### 3.2.2.2 Differential metabolite analysis

The table shows that 50 differential metabolites were counted, of which 17 cases were up-regulated and 33 cases were down-regulated in LC group compared with SD group. Analysis of the differential metabolites revealed that stearidonic acid was downregulated in the LC group. The ROC curve showed that the AUC value of stearidonic acid was 0.867, which can be used as a potential biomarker, and stearidonic acid is involved in the metabolism of α-linolenic acid. Differential metabolites interact within the organism, forming different pathways. All metabolites were annotated in the KEGG database, and the results were the same as those of group C *vs* D. The KEGG database annotation of differential metabolites showed that the pathways involved in differential metabolites included steroid hormone biosynthesis, folate biosynthesis, phosphonate and phosphinate metabolism, neomycin, kanamycin, gentamicin biosynthesis, and linoleic acid metabolism.

#### 3.2.3 Metagenomic and metabolomic joint analysis

##### 3.2.3.1 Strain-functional gene-metabolite association analysis

When strain-functional gene-metabolite association analysis was carried out in the LC *vs*. SD group, it was found that the gut microbiota *Dorea* and P*arasporobacterium* were correlated with the metabolite 3-O-Mycarosylerythronolide and were related to the K10762 gene.

##### 3.2.3.2 Random forest classification analysis of metabolomic and metagenomic taxonomy abundance, metagenomic functional genes

Through **Supplementary Figures 9A, B**, it can be seen that metagenomic species abundance and metagenomic functional genes have better modeling effects than metabolomic, with AUC of 0.80 ± 0.24 and 0.90 ± 0.20, respectively.

### 3.3 Metagenomic and metabolomic results of C *vs* LC

We then studied the changes in the gut microbiota of patients with long PFS who progressed after treatment.

#### 3.3.1 Metagenomic results

##### 3.3.1.1 Basic situation

In C *vs*. LC, the GO and KEGG functional annotations were the same as the results of the C *vs*. D group. There were no significant differences in the analysis of gene number differences, beta diversity analysis of functional genes, alpha diversity analysis of taxonomy, and beta diversity analysis of taxonomy between the C *vs*. LC groups, as in the C *vs*. D group.

##### 3.3.1.2 Differential analysis of functional genes

After treatment, the two groups showed significant differences in galactose metabolism, phenylalanine, tyrosine and tryptophan biosynthesis, pentose and glucuronate interconversions, riboflavin metabolism, tyrosine metabolism and novobiocin biosynthesis.

##### 3.3.1.3 Differential analysis of taxonomy

**Figure 9:**
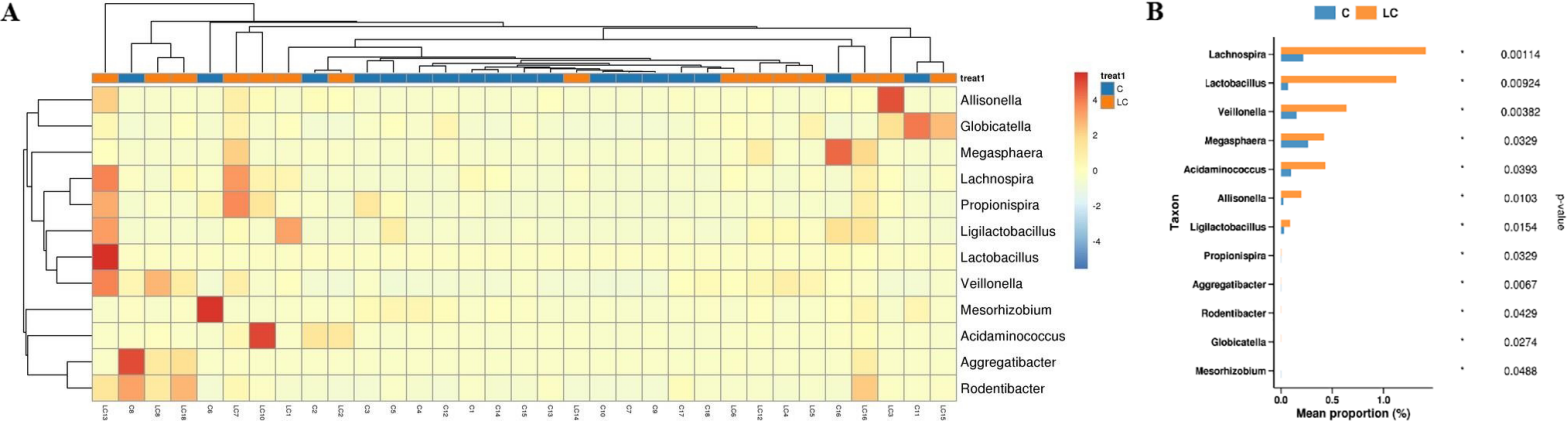
**(A)** Genus-level heat map on different species abundance in C vs LC group **(B)** Histogram of differential species abundance by genus level rank sum test in C vs LC group.

As shown in **Figures 9A, B**, in the patients with long PFS group, after chemotherapy combined with immunotherapy, the numbers of *Lachnospira, Lactobacillus, Veillonella, Megasphaera, Aminococcus*, and *Allisonella* increased compared with the baseline. At the species level, the main species were *Dialister_massiliensis, Lachnospira pectinoschiza, Dialister_sp._CAG_357*, and *Eubacterium.*

##### 3.3.1.4 Random forest analysis of taxonomy

Random forest analysis of species showed that *Veillonella, Lachnospira, Aggregatibacter, Lactobacillus, Allisonella, Butyricicoccus, Dialister*, and *Staphylococcus* were more important for sample classification.

#### 3.3.2 Metabolomic results

##### 3.3.2.1 PCA

After chemotherapy combined with immunotherapy, fecal metabolites in patients with long PFS showed significant changes, and the confidence circle of metabolites after treatment had a tendency to separate.

##### 3.3.2.2 Differential metabolite analysis

After the analysis, 186 metabolites were found to have significant differences after treatment, of which 12 were up-regulated and 174 were down-regulated. Leukotriene B4(LTB4) is a representative differential metabolite, and the AUC of LTB4 in the ROC curve analysis was 0.844, which can be used as a potential biomarker. In the KEGG functional annotation and enrichment analysis of differential metabolites, it was found that the pathways involved in the differential metabolites included steroid hormone biosynthesis, bile secretion, neomycin, kanamycin, gentamicin biosynthesis, monobactam biosynthesis, protein digestion and absorption, arachidonic acid metabolism, hormone biosynthesis, and ABC transporters.

#### 3.3.3 Metagenomic and metabolomic joint analysis

##### 3.3.3.1 Comparative multicohort downscaling analysis

**Figure 10:**
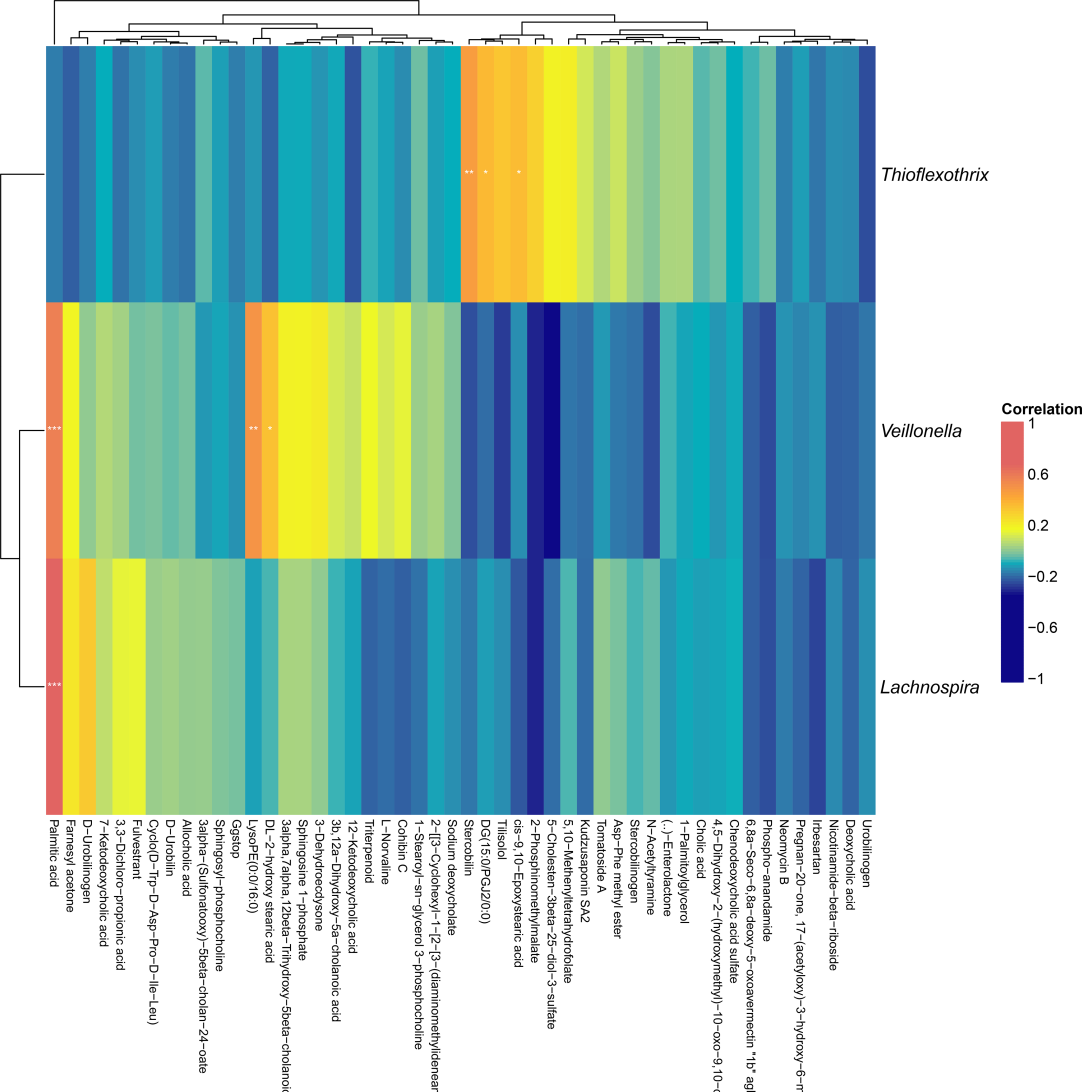
Heatmap of correlation analysis between differential metabolites and taxonomy abundance in C vs LC group.

After anti-tumor therapy, we found that the confidence circle of both gut microbiota and metabolites in patients with long PFS during PCA analysis tended to separate from the baseline (**Supplementary Figure 10A**, **Figure 10B**).

##### 3.3.3.2 Correlation analysis of differential metabolites and taxonomy abundance

In the heat map analysis of the correlation between differential metabolites and species abundance (**Figure 10**), we found that *Veillonella* and *Lachnospira* were positively correlated with palmitic acid.

##### 3.3.3.3 Random forest classification analysis of metabolomic and metagenomic taxonomy abundance, metagenomic functional genes

Through random forest analysis of metabolomic and metagenomic species abundance and metagenomic functional genes, we found that metagenomic species abundance and metagenomic functional genes had good modeling effects, andxtheir AUC were 0.80 ± 0.16 and 0.86 ± 0.16, respectively, and itxwas not recommended to combine and model with metabolomics.

### 3.4 Metagenomic and metabolomic results of D *vs* SD

After analyzing the feces of patients with long PFS before and after treatment, we again analyzed the feces of patients with short PFS before and after treatment.

#### 3.4.1 Metagenomic results

##### 3.4.1.1 Basic situation

In D *vs*. SD, the GO and KEGG functional annotations were the same as the results of the C *vs*. D group. There were no significant differences in the analysis of gene number differences, beta diversity analysis of functional genes, alpha diversity analysis of taxonomy, and beta diversity analysis of taxonomy between the D *vs*. SD groups, as in the C *vs*. D group.

##### 3.4.1.2 Differential analysis of taxonomy

**Figure 11:**
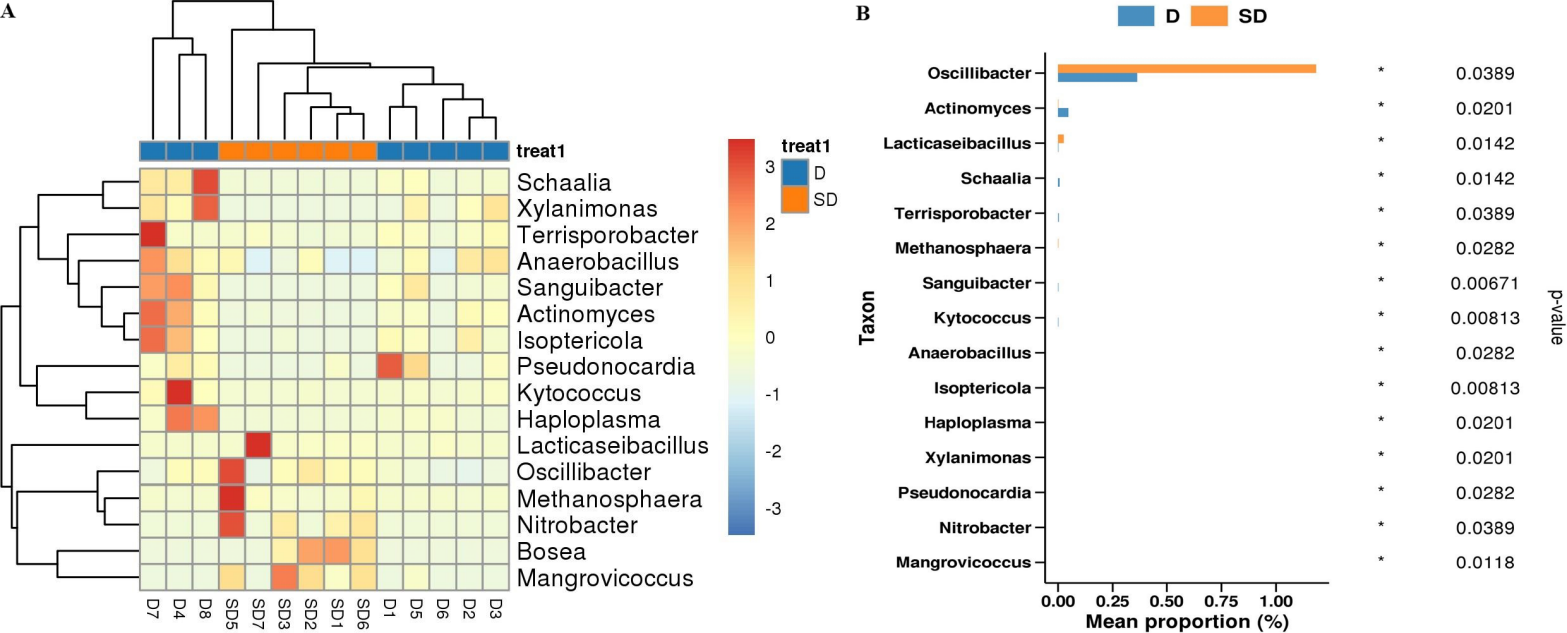
**(A)** Genus-level heat map on different species abundance in D vs SD group **(B)** Histogram of differential species abundance by genus level rank sum test in D vs SD group.

After treatment, we found differences in the abundance of *Oscillibacter, Actinomyces, Lacticaseibacillus, Schaalia, Anaerobacillus, Methanosphaera, Isoptericola* (**Figure 11A**). Among them, *Oscillibacter* and *Lacticaseibacillus* were highly expressed in SD, whereas *Actinomyces* and *Schaalia* were expressed at low levels in SD (**Figure 11B**). The abundance of differential species was dominated by *Schaalia, Actinomyces*, and *Granulicatella bacteria* at the species level.

##### 3.4.1.3 Random forest analysis of taxonomy

Random forest analysis revealed that *Schaalia, Methanosphaera, Sanguibacter, Actinomyces, Yersinia, Oscillibacter, Granulicatella* and *Lacticaseibacillus* were highly important for sample classification.

#### 3.4.2 Metabolomic results

##### 3.4.2.1 Differential metabolite analysis

By differential metabolite analysis, a total of nine differential metabolites were found, and all of them were downregulated. The pathways involved in differential metabolites were phenylalanine metabolism, riboflavin metabolism, ubiquinone and terpenoid quinone biosynthesis. Among the differential metabolites, trans-cinnamic acid can be used as a potential biomarker, with an AUC value of 0.792 in the ROC curve, and is involved in phenylalanine metabolism, ubiquinone, and other terpenoid-quinone biosynthesis.

#### 3.4.3 Metagenomic and metabolomic joint analysis

##### 3.4.3.1 Comparative multicohort downscaling analysis

PCA revealed a trend of separation of gut microbiota confidence circles and their KEGG confidence circles in the short PFS group before and after anti-tumor treatment (**Supplementary Figure 11A**, **Figure 11B**).

##### 3.4.3.2 Strain-functional gene-metabolite association analysis

**Figure 12:**
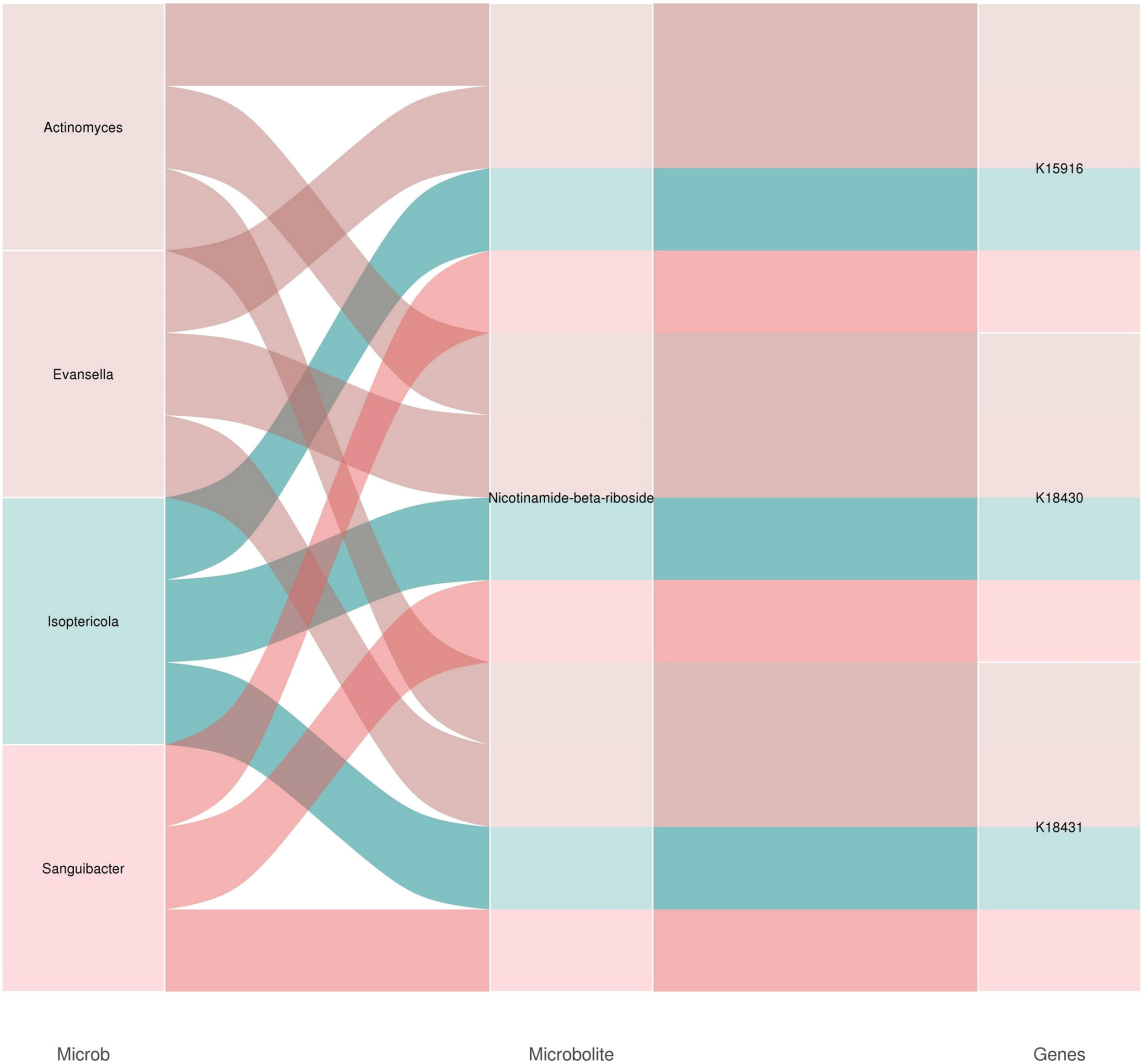
Strain-functional gene-metabolite association analysis sankey plot in D vs SD group.

By analyzing the Sankey plot of the strain- functional gene-metabolite association (**Figure 12**), we found that *Actinomyces, Evansella, Isoptericola*, and *Sanguibacter* were correlated with the metabolites nicotinamide-β-riboside, and were correlated with the functional genes of k15916, k18430, and k18431.

##### 3.4.3.3 Random forest classification analysis of metabolomic and metagenomic taxonomy abundance, metagenomic functional genes

As in the previous analyses, the modeling effect of random forest analysis based on metagenomic species abundance and metagenomic functional genes was good (AUC values: 0.99 ± 0.00 (both)), but the modeling effect of random forest analysis based on metabolites in the short PFS group was poor, with an AUC of 0.40 ± 0.49.

### 3.5 Metagenomic and metabolomic results of C+D *vs* LC+SD

Finally, we conducted metagenomic and metabolomic analyses of the feces of all patients before and after treatment to reveal the changes in the gut microbiota in the whole population.

#### 3.5.1 Metagenomic results

##### 3.5.1.1 Basic situation

In C+D *vs*. LC+SD, the GO functional annotation and KEGG functional annotation were the same as the results of the C *vs*. D group. There were no significant differences in the analysis of gene number differences, beta diversity analysis of functional genes, alpha diversity analysis of taxonomy, and beta diversity analysis of taxonomy between the C+D *vs*. LC+SD groups, as in the C *vs*. D group.

##### 3.5.1.2 Differential analysis of functional genes

Analysis of functional genes between groups showed differences in ABC transporters, purine metabolism, ribosome, galactose metabolism, glycine, serine and threonine metabolism, phenylalanine, tyrosine and tryptophan biosynthesis before and after treatment. The expression of ABC transporters and galactose metabolism was higher at baseline than after treatment, and purine metabolism and ribosome expression after treatment were higher than those before treatment (**Supplementary Figure 12**).

##### 3.5.1.3 Differential analysis of taxonomy

**Figure 13:**
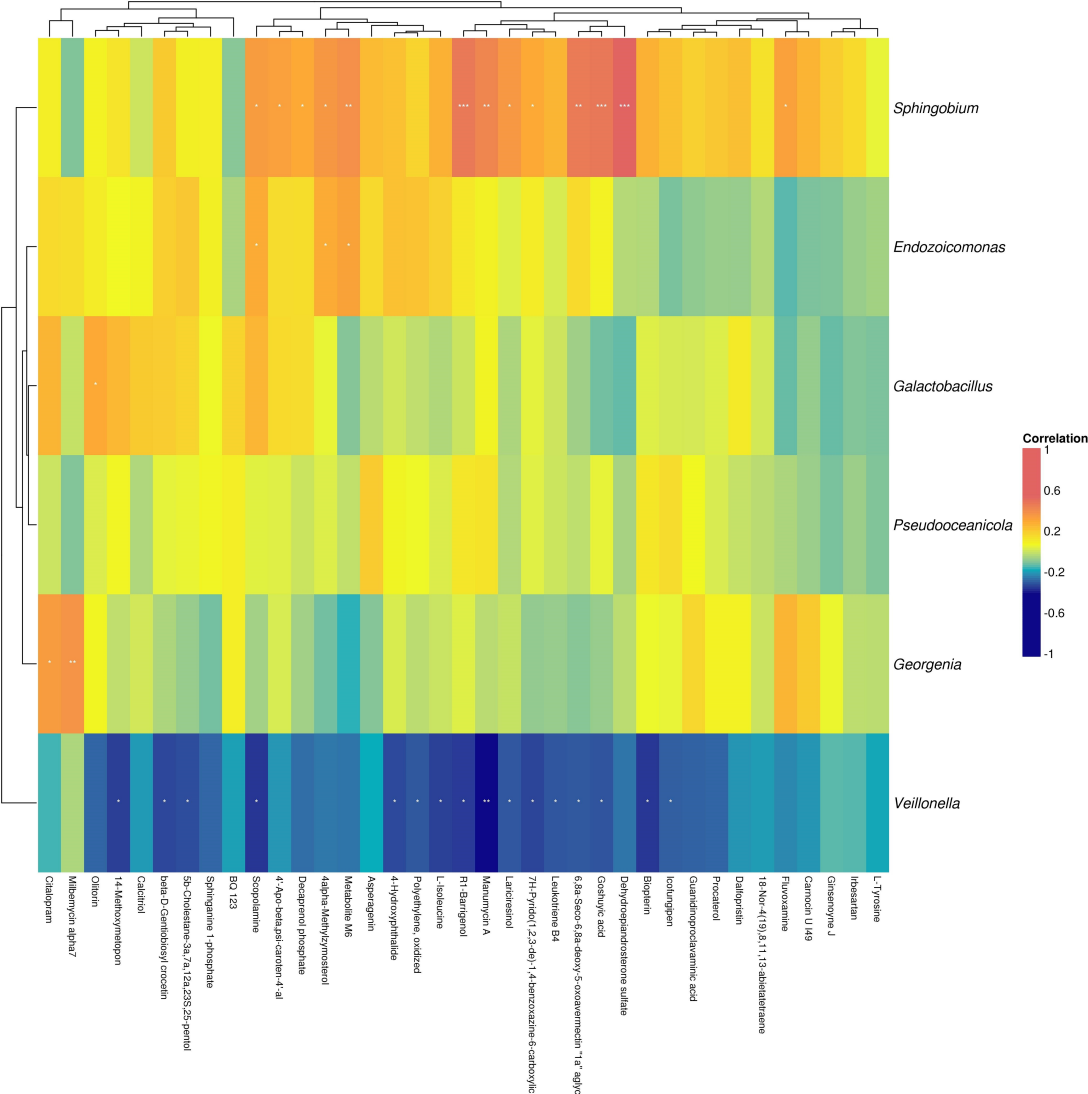
Heatmap of correlation analysis between differential metabolites and taxonomy abundance in C+D vs LC+SD group .

Compared with the baseline, the different species after immunotherapy combined with chemotherapy were mainly *Lachnospira, Ligilactobacillus, Veillonella, Allisonella*, and *Lacticaseibacillus*, and these bacteria were more enriched in patients after anti-tumor therapy (**Supplementary Figure 13A**, **Figure 13B**). At the species level, *Lachnospira, Bifidobacterium, Eubacterium* and *Dialister bacteria* showed the most obvious differences and were enriched in patients after treatment.

##### 3.5.1.4 Random forest analysis of taxonomy

Random forest analysis revealed that *Trichospirillum, Schaalia, Lacticaseibacillus, Pectobacterium, Veillonella, Dorea, Ligilactobacillus, Staphylococcus*, and *Akkermansia* were more important for the classification of samples.

#### 3.5.2 Metabolomic results

##### 3.5.2.1 PCA

Surprisingly, in the analysis of LC *vs*. SD and C *vs*. LC, the metabolite PCA analysis showed a separation trend in the metabolite confidence circle between the two groups, whereas in the analysis of C *vs*. D, D *vs*. SD, and C+ D *vs*. LC +SD, the metabolite PCA analysis showed no separation trend in the metabolite confidence circles between the two groups.

##### 3.5.2.2 Differential metabolite analysis

In the analysis of differential metabolites, 37 differential metabolites were identified, all of which were downregulated metabolites and none were upregulated metabolites. KEGG functional annotation and enrichment analysis of differential metabolites showed that differential metabolites were mainly involved in bile secretion, central carbon metabolism in cancer, mineral absorption, PPAR signaling pathway, hormone biosynthesis, neuroactive ligand-receptor interaction, and aminoacyl-tRNA biosynthesis. Among the differential metabolites, L-tyrosine, L-isoleucine, and LTB4 (**Supplementary Figure 14**) were represented, and their AUC showed in ROC curve analysis were 0.751, 0.797, and 0.803, respectively, indicating that they could be used as potential biomarkers. In the differential metabolite KEGG function annotation pathway diagram, L-tyrosine was involved in tyrosine metabolism, ubiquinone and other terpenoid-quinone biosynthesis, thiamine metabolism and monobactam biosynthesis. L-isoleucine was involved in the biosynthesis and degradation of valine, leucine, isoleucine, and aminoacyl-tRNA biosynthesis. LTB4 was involved in serotonergic synapses and neuroactive ligand-receptor interactions.

#### 3.5.3 Metagenomic and metabolomic joint analysis

##### 3.5.3.1 Correlation analysis of differential metabolites and taxonomy abundance

By analyzing the heat map of the correlation between differential metabolites and species abundance (**Figure 13**), we found the differential gut microbiota of *Veillonella* previously identified by metagenomic, and found that it was negatively correlated with the differential metabolites leukotriene B4 and L-isoleucine.

##### 3.5.3.2 Random forest classification analysis of metabolomic and metagenomic taxonomy abundance, metagenomic functional genes

The ROC curve analysis of metabolomic, metagenomic species abundance and metagenomic functional genes showed that the AUC value of metabolites in the C+D *vs*. LC+SD group was higher than that in the previous analyses, and was 0.73 ± 0.16 (**Supplementary Figure 15**), which was the same as the modeling effect of LC *vs*. SD. Therefore, we can predict whether the LC or SD group, or the C+D or LC+SD group by metabolites. Metabolite modelling was not effective in the C *vs*. D group. Can we understand here that as the anti-tumor treatment proceeds, metabolites play an increasingly important role in it.

## 4 Discussion

Although SCLC accounts for only approximately 15-20% of LC, it is highly malignant and invasive. Most SCLC patients are in the extensive stage when first diagnosed, and systemic chemotherapy has been the preferred treatment option in the past. Although the initial treatment is highly effective, it is prone to drug resistance, progresses rapidly, and has a poor overall prognosis. In recent years, immunotherapy, such as PD-1/PD-L1 antibody drugs, have significantly improved the survival prognosis of patients with ES-SCLC. The IMpower133 and CASPIAN studies showed that immunotherapy combined with chemotherapy achieved breakthrough progress in OS in the first-line treatment of patients with ES-SCLC. As a result, the FDA approved the PD-L1 monoclonal antibodies Atezolizumab and Durvalumab are used in combination with carboplatin and etoposide for the first-line treatment of patients with ES-SCLC (39, 40). Therefore, the current standard first-line treatment for ES-SCLC is chemotherapy combined with immunotherapy. However, the patient population and the therapeutic efficacy of immune combination chemotherapy are limited. Therefore, how to screen potential benefiting patients and further improve the efficacy is an urgent clinical issue.

The gut microbiota has been implicated as a hazard or preventive factor for various diseases, including cancer (41–43). Through in-depth studies of the gut microbiota, researchers have proposed several ways in which the gut microbiota affects distal organs, such as the microbiota-gut-lung axis, the microbiota-gut-brain axis and the microbiota-gut-liver axis (44, 45). The mechanism underlying carcinogenesis caused by gut microbiota includes its producing toxic metabolites, inducing inflammation milieu, and suppressing antitumor immunity, which leads to genomic instability, DNA damage, and immune escape in tumor tissue (46, 47). In recent years, the link between gut microbiota and tumor immunotherapy has been widely recognized. Gut microbiota influences anti-tumor immunity in a variety of ways. The intricate crosstalk between the gut microbiota and the host immune system initiates at the intestinal epithelial. When pathogen-associated molecular patterns (PAMPs) derived from gut microbes bind to pattern recognition receptors (PRRs) on the surface of innate immune cells and intestinal epithelial cells, antigen-presenting cells such as dendritic cells (DCs) are activated. This triggers the abundant production of pro-inflammatory cytokines—including tumor necrosis factor-α, IL-12, and IFN—thereby promoting Th1/Tc1 immune responses against cancer. DCs are activated and migrate from the gut-associated lymphoid tissues (GALT) to the mesenteric lymph nodes, further promoting the activation of naive CD4+ and CD8+ T cells (48). Lam et al. proposed that the gut microbiota can reprogram intratumoral mononuclear phagocytes into immunostimulatory monocytes and DCs by modulating the NK cell- DC axis and type I interferon signaling, thereby reshaping the innate immune landscape in the tumor microenvironment. When the gut microbiota was depleted, these mononuclear phagocytes tended to differentiate into pro-tumor macrophages (49). Antigens from certain gut bacteria can induce antigen-specific immune responses and cross-reacts with tumor-associated antigens (TAA) to inhibit tumor progression. For example, Enterococcus hirae produces antigens with epitopes similar to those of TAA, thereby inducing CD4+ T-helper type 1 (Th1) cell responses in patients with advanced LC receiving chemotherapy combined with immunotherapy (50). Pattern recognition receptors (PRRs), such as lipopolysaccharide (LPS), produced by gut microbiota can activate Toll-like receptor 4 (TLR4), which induces a series of inflammatory reactions that affects the development of liver cancer (51). TLR9 has also been found to be involved in the anti-tumor immune response of liver cancer mediated by the gut microbiota (52).

As research on gut microbiota advances, the intratumoral microbiota has also gained scientific attention. Intratumoral microbiota might modulate anti-tumor immunity in many ways, including activating the PERK and STING pathways, promoting lymph node maturation, and enhancing cellular immunity mediated by bacterial peptides. Despite these beneficial effects, the intratumoral microbiota can also hinder anti-tumor immunity and reduce the efficacy of cancer immunotherapy. When mucosal barriers are compromised, Fusobacterium *and Bacteroides fragilis* drive intestinal tumorigenesis by generating reactive oxygen species (ROS), which regulate localized inflammation but can impair T cell function and anti-tumor responses (53). The immunosuppressive microenvironment shaped by long-term antigen exposure and microbial metabolites can lead to T cell exhaustion, which is characterized by loss of effector function and upregulation of PD-1 expression. This exhausted state reduces T cell responsiveness to immunotherapies (53). Intratumoral bacteria can also stimulate myeloid cells to produce MyD88-dependent IL-23and IL-1β, which activate γδ T cells to secrete IL-17, thereby promoting B cell infiltration and tumor development (53).

In addition, metabolites of the gut microbiota play an important role in modulating anti-tumor immunity. For example, butyrate produced by Faecalibacterium prausnitzii has been linked to an improved clinical response to anti-tumor therapy with immunotherapy (54). The antitu anti-tumor mor immune response involves different immune cell subsets, including CD8+ T cells, antigen-presenting cells (APCs), CD4+ T regulatory cells (Tregs), CD4+ Th1 cells, and myeloid-derived suppressor cells (MDSCs) (55–57). Specifically, interferon-γ-producing effector CD8+ T cells were induced by supplementation with a consortium of 11 bacterial strains, thereby enhancing anti-tumor immunity (58). Mechanistically, the increase in T cells is associated with cell expansion, bacteria-mediated chronic recruitment, and bacterial antigen-induced differentiation.

These findings led us to believe that the regulation of gut microbiota may be a potential strategy to improve the response to tumor therapy. In order to develop gut microbiota-based antitum anti-tumor or therapies, the identification of target gut bacterial species is required (59). Therefore, we studied the gut microbiota of patients with SCLC with long and short PFS before and after treatment.

### 4.1 The analysis of C *vs* D

In the analysis of differential species between groups, *Streptococcus* was highly expressed in the short PFS group, and *Actinomyces* was highly expressed in the long PFS group. This is consistent with previous studies reporting that Streptococcus is an unfavorable genus, whereas *Actinomyces* is a beneficial genus. Some studies have reported the presence ofunique intratumoral microbiota in breast cancer (BC) tissues, including *Streptococcus, Lactobacillus* and *staphylococcus*, which are potential factors leading to tumor metastasis (60). *Streptococcus* has also been linked to oral cancer (61, 62). In colorectal cancer (CRC), *Streptococcus, Lactococcus, Fusobacterium, Prevotella*, and *Bacteroides* are more abundant in tumor tissues than in normal tissues (63). In a study of head and neck squamous cell carcinoma (HNSCC), *Actinomyces* was significantly reduced and *Parvimonas* was elevated compared to those in normal tissue (64). Studies have shown that *Firmicutes* and *Actinomyces* are enriched in responders to fecal microbiota transplantation (FMT) combined with PD-1 blockade therapy (65). A research team used Mendelian randomization to study whether gut microbiota has a causal relationship with cancer. The results showed that the phylum *Actinobacteria* and class *Actinobacteria* are risk factors for LC and breast cancer but are protective factors for oral cavity cancer (66). The study also suggests that different species may have different effects on the tumor microenvironment (23). Recent research has found that the abundance of A. odontolyticus is significantly increased in the feces of early-stage CRC patients, clarifying a new mechanism: A. odontolyticus can drive colorectal dysplasia by secreting membrane vesicles. These vesicles induce ROS production and mitochondrial dysfunction, ultimately leading to DNA damage and subsequent cellular transformation (67). A. odontolyticus can also activate the NF-κB pathway, and this activation effect is mediated by its lipoteichoic acid specifically binding to the TLR2 receptor.

Among the differential metabolites detected, indirubin-3’-monoxime inhibited CDK at higher concentrations, reversibly inhibiting the proliferation of multiple cell types and preventing the G2/M phase cycle. In our study, indirubin-3’-monoxime was higher in the long PFS group than in the short PFS group, and ROC curve analysis showed that it could be used as a potential biomarker. Indirubin-3’-monoxime induces cancer cell death. It has been previously reported to have inhibitory effects on human osteosarcoma and anti-proliferation and apoptosis-inducing effects in renal cell carcinoma (68, 69). Indirubin-3’-monoxime has been shown to have anti-tumor effects on LC through its apoptotic effect in mice (70). The combination of indirubin-3’-monoxime and thymoquinone has excellent therapeutic efficacy in LC models (71). These findings are consistent with our results, however, further research is needed on the role of indirubin-3’-monoxime in cancer.

According to the ROC curves of metabolomic and metagenomic species abundance and metagenomic functional gene modeling, metagenomic species abundance and metagenomic functional gene modeling were good. Therefore, in future clinical studies, we can predict the effect of immunotherapy by monitoring the fecal gut microbiota. Of course, a single gut microbiota is slightly thin, and we can make a comprehensive judgment by combining other tumor markers and prognostic indicators.

### 4.2 The analysis of LC *vs* SD

When comparative analysis was conducted on patients with long PFS and short PFS after treatment, that is, LC *vs*. SD, intergroup differential species analysis revealed that the genus *Roseburia* was highly expressed in the long PFS group, and at the species level, different species were reflected in the bacteria of *Eubacterium, Clostridiales, Candidatus* and *Roseburia.* The findings regarding the *Roseburia* and *Eubacterium* bacteria are consistent with previously reported results. *Roseburia* produces short-chain fatty acids (acetic, propionic, and butyric acids) that break down indigestible carbohydrates. Short-chain fatty acids are generally considered to play a variety of important roles in maintaining human health, such as acting as special nutritional and energy components of the intestinal epithelium, protecting the intestinal mucosal barrier, reducing the level of human inflammation, and enhancing gastrointestinal motility. Typical *Roseburia* strains can produce high levels of butyrate. These compounds are often involved in energy production and can protect the gut from pathogens and diseases. As an essential bacterial metabolite, butyrate concentration is negatively correlated with the incidence of CRC (72, 73). Current studies have shown that butyrate can inhibit tumor growth in mice and reverse immunosuppression caused by increased levels of PD-L1 and IL-10 in peripheral blood mononuclear cells and tumor cells in patients with gastric cancer (74). *Roseburia intestinalis (R.intestinalis)* produces butyrate, which directly enhances cytotoxic CD8+ T cell function through toll-like receptor 5 (TLR5)-dependent nuclear factor-κB (NF-κB) signaling (75). *R. intestinalis* also limits the growth of CRC tumors by regulating anti-tumor CD8+ T cell responses (75). Therefore, *R.intestinalis* is a promising probiotic supplement that has the potential to open a new avenue for improving treatment outcomes in patients with CRC, especially for those who are resistant to PD-1 therapy. *Eubacterium*, belonging to Firmicutes, is a relatively rich bacterium in the human gastrointestinal tract that can produce short-chain fatty acids, especially butyric acid. Several members of *Eubacterium* produce butyrate, which plays a key role in energy homeostasis, immune regulation, colonic motility, and suppression of intestinal inflammation (76). In addition, *Eubacterium* members have been shown to play key roles in promoting the production of anti-inflammatory molecules, bile acid and cholesterol transformation, preventing allergic airway inflammation, participating in oxalate catabolism, and reducing insulin secretion. Reduction or absence of *Eubacterium* has been shown to be associated with CRC, tumor prognosis, depression/fatigue, inflammatory bowel disease, type 2 diabetes, and obesity (76).

Among the differential metabolites, stearidonic acid was downregulated in the LC group. The ROC curve indicated that it could serve as a potential biomarker, and stearidonic acid was involved in α-linolenic acid metabolism. Stearidonic acid-enriched flax oil reduced the growth of human BC both *in vitro* and *in vivo* (77). The combination of omega-3 stearidonic acid and docetaxel enhances cell death compared to docetaxel alone in human prostate cancer cells (78).

### 4.3 The analysis of C *vs* LC

To gain a more detailed understanding of the changes in gut microbiota, we conducted metagenomic and metabolomic analyses of the feces of patients with long PFS before and after treatment, that is, C *vs*. LC. After chemotherapy combined with immunotherapy, the numbers of *Lachnospira, Lactobacillus, Veillonella, Megasphaera, Aminococcus*, and *Allisonella* increased compared to the baseline. A study analyzed the gut microbiota composition and serum metabolic profile of 30 LC patients at different stages and 15 healthy individuals and found that L-valine decreased with the progression of the disease. Correspondingly, the genus with the strongest association with L-valine, *Lachnospiraceae_UCG-006*, was reduced in the LC group (79). The microbiome of patients at different histological stages also changes from gastritis to precancerous lesions and then to gastric cancer, and the abundance of *Lachnospiraceae* increases with disease progression (80). In a previous study, 16S rRNA gene sequencing of paired lung tumors and remote normal samples from the same lung segment/lobe in 19 patients with NSCLC was performed (81). It was found that higher *Koribacteraceae* abundance in normal tissues was associated with increased recurrence-free (RFS) and disease-free survival (DFS). Higher abundances of *Ruminococcaceae, Lachnospiraceae*, and *Bacteroidaceae* were associated with lower RFS or DFS. Lung adenocarcinoma xenograft mice fed with *Lactobacillus acidophilus* solution showed a reduction in tumor volume and an increase in survival compared to treatment with cisplatin alone (82). It was found that *Lactobacillus rhamnosus (Probio-M9)* alone had no significant effect on tumor inhibition. However, in anti-PD-1 therapy, *Probio-M9* promotes the anti-tumor immune response to anti-PD-1 therapy by enhancing beneficial bacteria and inhibiting harmful bacteria (83). The compositional characteristics of lung flora have not yet been determined, but *Streptococcus, Veillonella* and *Megasphaera* may be key marker genera for the early diagnosis of LC (84, 85).

LTB4 is a representative differential metabolite that could be used as a potential biomarker. LTB4 is a leukocyte chemoattractant that plays a major role in the control of inflammatory responses. LTB4 is associated with the progression of pancreatic, hepatocellular, colorectal, and renal cancer (86–89). In the joint analysis of the correlation between differential metabolites and species abundance, *Veillonella* and *Lachnospira* were positively correlated with palmitic acid. It has been shown that palmitic acid can inhibit the proliferation and metastasis of prostate cancer cells by inhibiting the PI3K/Akt pathway (90). Studies have also shown that palmitic acid promotes gastric cancer metastasis (91). The role of palmitic acid in LC requires further exploration, especially when combined with *Veillonella* and *Lachnospira*.

### 4.4 The analysis of D *vs* SD

After analyzing the feces of patients with long PFS before and after treatment, we again analyzed the feces of patients with short PFS before and after treatment. After treatment, *Actinomyces* and *Schaalia* were expressed at low levels in SD. A preclinical study analyzing the impact of gut microbiota modulation on the growth of hepatocellular carcinoma showed that tumor growth was significantly inhibited when model mice were fed the probiotic mixture “Prohep”. Analysis of the gut microbiota of the model mice revealed significant enrichment of several gut microbiota, including *Oscillibacter*. An increase in *Oscillibacter* and *Prevotella* decreased the number of tumor-infiltrating Th17 cells (92, 93). In an analysis of the gut microbiota of patients with gastric cancer and gastrointestinal stromal tumors, it was found that the abundance of *Oscillibacter* and *Lactobacilliaceae* was lower in patients with tumors than in healthy controls (94). Paradoxically, another clinical study showed an increased abundance of *Oscillibacter, Roseburia, Bacteroides*, and *Ruminococcus* in the mucosal flora of CRC patients, but no mechanistic study was mentioned, so this finding needs to be further validated (95, 96). At present, there are few studies on the role of *Schaalia* in cancer.

Among the differential metabolites, trans-cinnamic acid may serve as a potential biomarker and is involved in phenylalanine metabolism. Trans-cinnamic acid has a broad spectrum of biological activities and anti-inflammatory, antioxidant, and anticancer properties (97, 98). It has been reported to have antiproliferative activity against melanoma cells and LC cells (99, 100). In addition, trans-cinnamic acid and its analogues have been detected as gut microbe−derived metabolites that exert various biological effects in the colon (101, 102). Studies have shown that trans-cinnamic acid can inhibit histone deacetylase and its anti-tumor effect on xenografts of colon cancer in athymic mice (103). Studies have been conducted to design and optimize trans-cinnamic acid-loaded PLGA nanoparticles and to evaluate their inhibitory effect on epithelial-mesenchymal transformation (EMT) in triple-negative BC (104). The results demonstrate the ability of PLGA nanoparticles to load trans-cinnamic acid and potentially enhance their anti-tumor efficacy in triple-negative BC by inhibiting EMT. We hope that this discovery will provide new insights for future scientific studies.

### 4.5 The analysis of C+D *vs* LC+SD

Finally, we conducted metagenomic and metabolomic analyses of the feces of all patients before and after treatment to reveal the changes in gut microbiota in the whole population, that is, C+D *vs*. LC+SD. Compared with baseline, at the species level, *Lachnospira, Bifidobacterium, Eubacterium* and *Dialister* bacteria showed the most obvious differences, and were more enriched in patients after treatment. Studies have shown that *Bifidobacterium* is closely related to the efficacy of immunotherapy, and its mechanism is mainly to promote the activation of dendritic cells in homeostasis, and then improve the effector function of tumor-specific CD8+T cells (23, 105, 106). Moreover, only specific *Bifidobacterium* strains have synergistic effects with anti-PD-1 to reduce tumor growth, while other *Bifidobacterium* strains have no synergistic effects with anti-PD-1. *Bifidobacterium* sp*ecies B. longum* has been reported to be enriched in NSCLC patients who respond to anti-PD-1 therapy and have higher levels of natural killer (NK) T cells and memory CD8+ T cells in their blood (107). In another clinical study, researchers found that melanoma patients who responded to PD-1 blockade therapy had more Bifidobacterium than those who did not respond to PD-1 blockade therapy (21). Even considering the lack of direct evidence and mechanism research, these findings indicate that *Bifidobacterium* is beneficial in anti-tumor immune responses. On the other hand, *Bifidobacterium* is also associated with irAEs, with more *Bifidobacterium* present in the gut microbiota of patients who did not suffer from irAEs (22). The abundance of *Bifidobacterium* was lower in patients with colitis-type irAEs (108). The researchers observed that *Bifidobacterium* strains produce inosine, a metabolite with multiple functions in the body that plays a key role in immune activation. Inosine greatly increases the expression of tumor antigens and induces cytotoxic immune cells to quickly recognize and eliminate tumor cells. This study showed that the presence of inosine increased the levels of TNF-α and IFN-γ, which further increased tumor antigen presentation and T cell activity (52). In addition, inosine is also involved in macrophage-mediated antibody production (34). A study aimed at investigating and characterizing the gut microbiota composition of patients with esophageal cancer showed that the abundance of *Dialister* and *Prevotella* was significantly reduced relative to that in healthy individuals (109). Another study showed that the genus *Dialister pneumosintes* was highly enriched in gastric cancer (110). *Dialister pneumosintes* and *Mobiluncus curtisi* were identified as significantly more common in vaginal samples of endometrial cancer and may be considered as potential endometrial cancer cofactors that promote or stimulate carcinogenesis (111).

Among the differential metabolites, L-tyrosine, L-isoleucine, and LTB4 were represented, which could be used as potential biomarkers. In the differential metabolite KEGG function annotation pathway diagram, L-tyrosine is involved in tyrosine metabolism, ubiquinone and other terpenoid-quinone biosynthesis, thiamine metabolism, and monobactam biosynthesis. L-isoleucine is involved in the biosynthesis and degradation of valine, leucine, and isoleucine, and aminoacyl-tRNA. LTB4 is involved in serotonergic synapses and neuroactive ligand-receptor interactions. In the analysis of C *vs*. LC, it was also found that LTB4 and L-isoleucine could be used as potential markers, but L-isoleucine is not a differential metabolite. LTB4 was reduced in the post-treatment samples both in the analysis of C *vs*. LC and in the analysis of C+D *vs*. LC+SD. LTB4 is a leukocyte chemoattractant that plays an important role in controlling inflammatory responses and is associated with cancer progression (112). LTB4 is involved in tumor progression by activating its receptor LTB4 receptor type 2 (113). Recent findings have indicated that the LTB4/BLT1 axis can achieve different outcomes in tumor progression (114). Deletion of BLT1 alleviated neutrophil inflammation and tumor promotion in a silicosis-induced inflammation-promoted mouse lung tumor model. In contrast, in spontaneous models of intestinal tumorigenesis, loss of BLT1 leads to microbiome alterations, defective mucosal host responses, and bacteria-dependent colon tumor progression (115). In addition, BLT1-mediated recruitment of CD8+T cells has been shown to be critical for initiating anti-tumor immunity in many xenograft models, and is essential for effective PD-1-based immunotherapy. The anti-tumor effects of the probiotics Clostridium butyricum and Bacillus subtilis on CRC progression have been demonstrated in mice (74).

In the combined analysis, by analyzing the heat map of the correlation between differential metabolites and species abundance, a negative correlation was found between the differential gut microbiota Veillonella and the differential metabolites leukotriene B4 and L-isoleucine. Random forest analysis revealed that the metabolite modeling effect of the C+D *vs*. LC+SD group and LC *vs* SD group was better. Metabolite modeling was poor at baseline. Thus, as anti-tumor treatment proceeds, metabolites play an increasingly important role.

### 4.6 Clinical application of the gut microbiota

To date, the gut microbiota has been used in cancer treatment in three main ways: dietary intervention, oral probiotics, and FMT. Dietary changes can rapidly alter the composition of gut microbiota (116). This means that this could be a safe and simple way to regulate the gut microbiota in patients with immunotherapy (117). The anti-tumor effects of probiotics *Bacillus subtilis* and *Clostridium butyricum* on CRC progression have been demonstrated in mice (118). Another prospective clinical study indicated that long-term intake of high-dose yogurt (containing *Lactobacillus bulgaricus* and *Streptococcus thermophilus*) significantly reduced the risk of CRC (76). FMT has been widely used in the treatment of many diseases, including cancer. Previous animal experiments have revealed that feeding mice with specific bacteria or FMT can improve their sensitivity to immunotherapy (65, 119). A phase I clinical trial was conducted in patients with anti-PD-1 refractory metastatic melanoma, in whom 3 of 10 patients went into remission after FMT intervention (119). Therefore, converting several specific bacteria into drugs may be a new clinical adjunct for cancer immunotherapy.

### 4.7 Limitations of the study

In contrast to observational studies, randomized controlled trials of the gut microbiota may help establish causal relationships. However, due to the influence of objective factors such as research methods and technology, there are still great limitations in the screening of strains involved in prognosis judgment and early diagnosis. Therefore, most current research findings are based on observations of the components and changes in gut microbiota in patients’ feces, as well as the results of experiments involving transplanting gut microbiota into germ-free mice. These results are affected by multiple factors such as antibiotic use and diet (120–122). Regarding the differential metabolites indirubin-3’-monoxime, leukotriene B4, and trans-cinnamic acid, although we have found evidence supporting their potential as biomarkers, our correlation analysis between differential metabolites and species abundance did not reveal any associations with differential gut microbiota. These results should be considered exploratory, as the current findings are based on human gut microbiota. We plan to validate these results in mouse models in subsequent studies. Because the gut microbiota is considered to be highly dynamic, the causal relationship between the gut microbiota and cancer has been an unresolved issue in the field. Therefore, it is important to explore the causal relationship between the gut microbiota and cancer.

However, our study had several limitations. First, most of the patients in our study were from Dalian, which may have led to bias and affected generalizability. Second, the strict screening conditions and lower incidence of SCLC compared to NSCLC resulted in a smaller sample size. In our study, the insufficient sample size may lead to statistical bias. We hope to expand the sample size in future studies, conduct further research through multicenter collaborations, and conduct more aspects of the study, including the gut microbiota of NSCLC. Third, the current analysis is mainly based on fecal samples, but the microbes in fecal samples do not fully reflect the real ecosystem in the gut. Therefore, the detection of the dynamic changes in the human gut microbiota after immunotherapy treatment remains a problem. We are also interested in the study of intra-tumor microbiota, and the next step will be to study intra-tumor microbiota.

## 5 Conclusions

The main issues with the use of immunotherapy drugs are the low response rate and the possibility of irAEs such as immune myocarditis and pneumonia, which may have negative or even fatal effects on patients. Therefore, it is crucial to optimize immunotherapy to reduce irAEs and improve its efficacy. Several basic and clinical studies have reported the effects of the gut microbiome on tumor immunotherapy. Colonization of cancer-associated microbiota induces a series of inflammatory responses, increases the expression of TNF-α, IFN-γ, and IL-1β, and activates T cells. Thus, combined bacterial therapy can overcome patient’s resistance to immunotherapy. In the future, we will further investigate the mechanisms of action between the different gut microbiota and the different metabolites that we have discovered and cancer. Notably, not all gut microbiota that can elicit an immune response are beneficial. Maintaining the abundance of beneficial microbiota may also promote the use of gut microbiota in the cancer therapy. It is also possible to intervene in the gut microbiota through the use of probiotics and prebiotics, diet, and FMT to modulate the subsequent immune responses.

## Data Availability

The data presented in the study are deposited in the NCBI and MetaboLights repository, accession number PRJNA1391873 and 239 MTBLS12382.
