# Supplementary figures and images for: Correlation between gut microbiota and their metabolites and the efficacy of chemotherapy combined with immunotherapy for extensive-stage small cell lung cancer
Source: Front Oncol. 2026 Mar 3;15:1683347. doi: 10.3389/fonc.2025.1683347 (PMC12992022; doi:10.3389/fonc.2025.1683347)

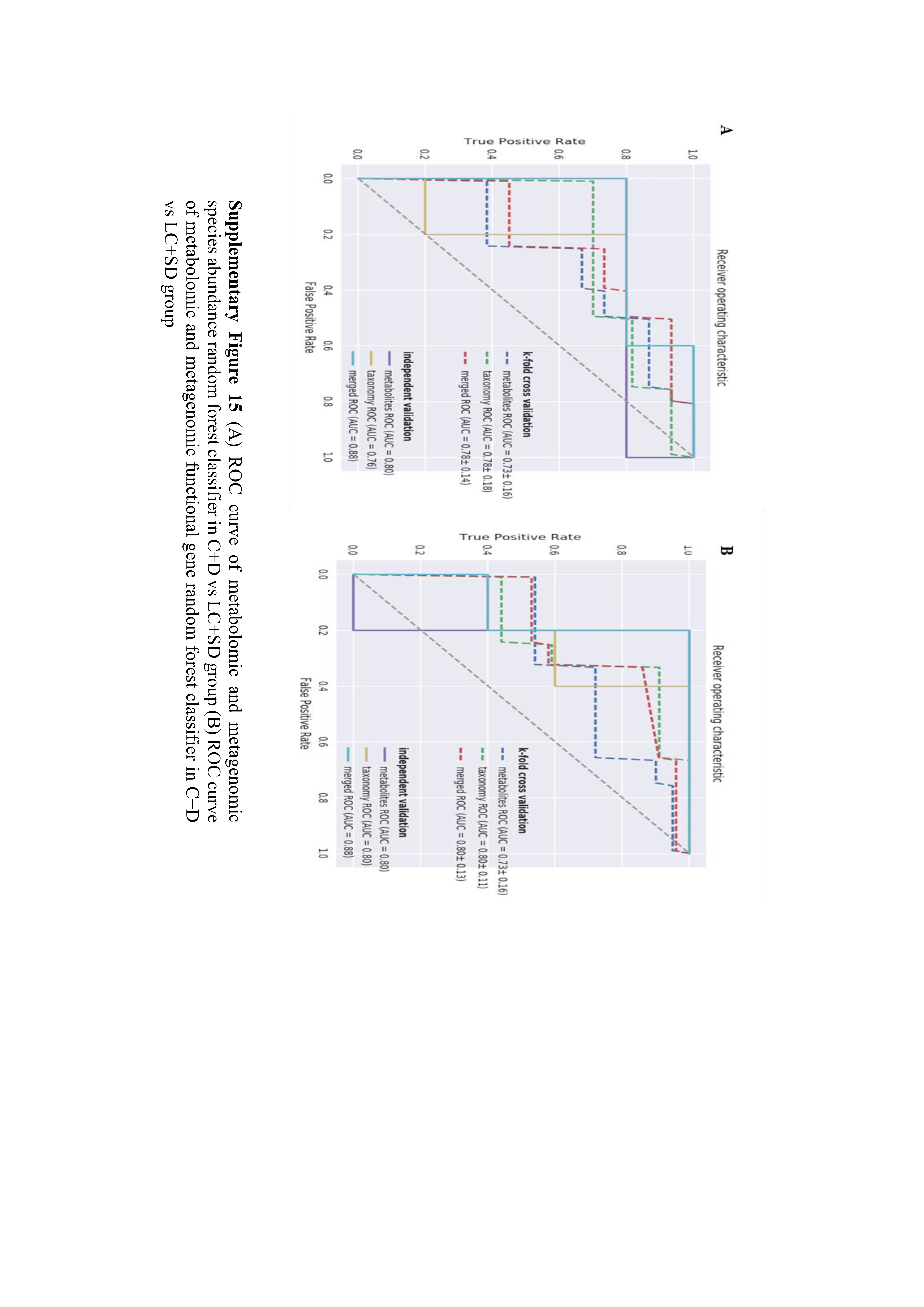

Supplement: Supplementary file 1 [file DataSheet1.zip › Supplementary Figure 15.jpg]

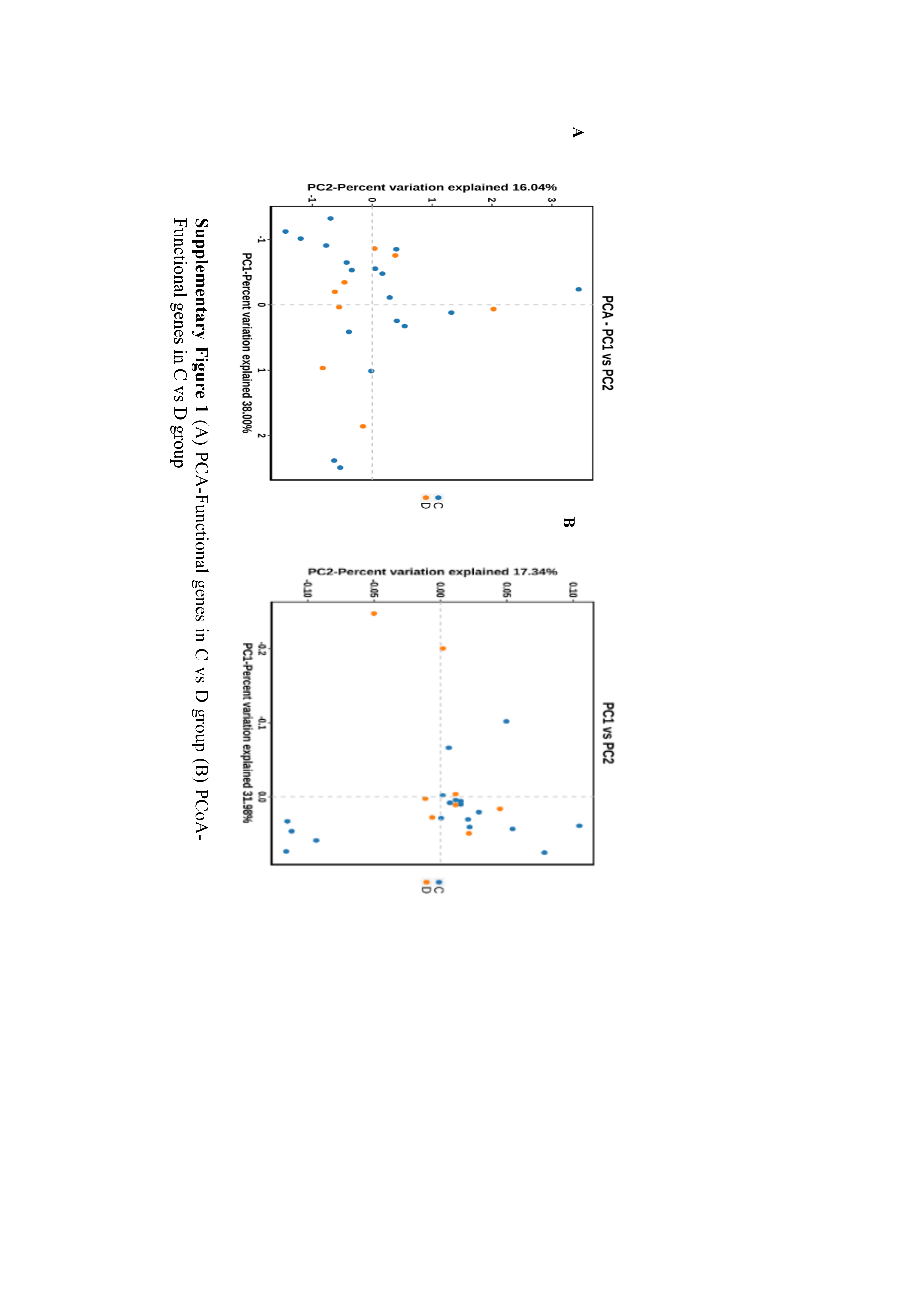

Supplement: Supplementary file 1 [file DataSheet1.zip › Supplementary Figure 1.jpg]

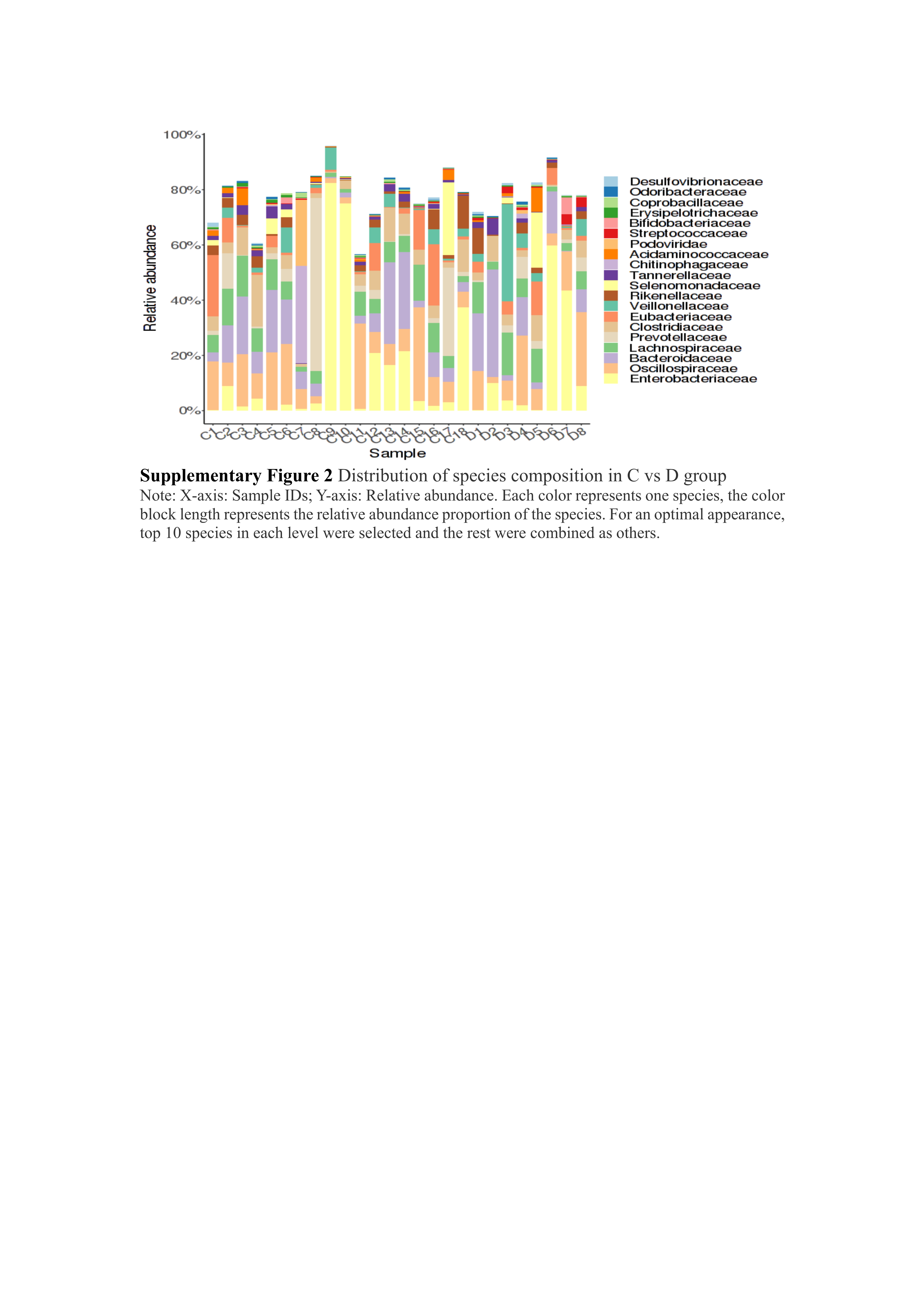

Supplement: Supplementary file 1 [file DataSheet1.zip › Supplementary Figure 2.jpg]

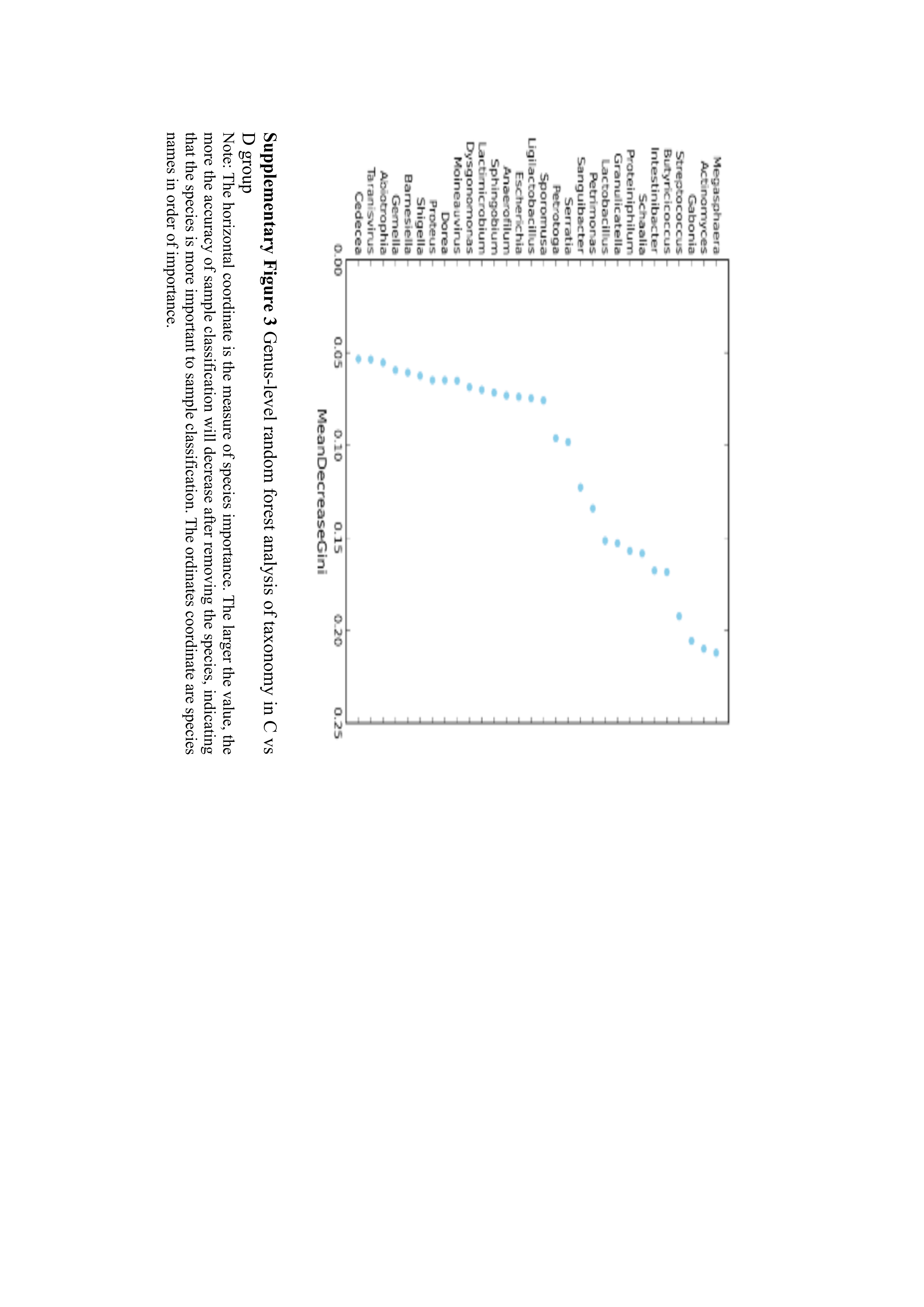

Supplement: Supplementary file 1 [file DataSheet1.zip › Supplementary Figure 3.jpg]

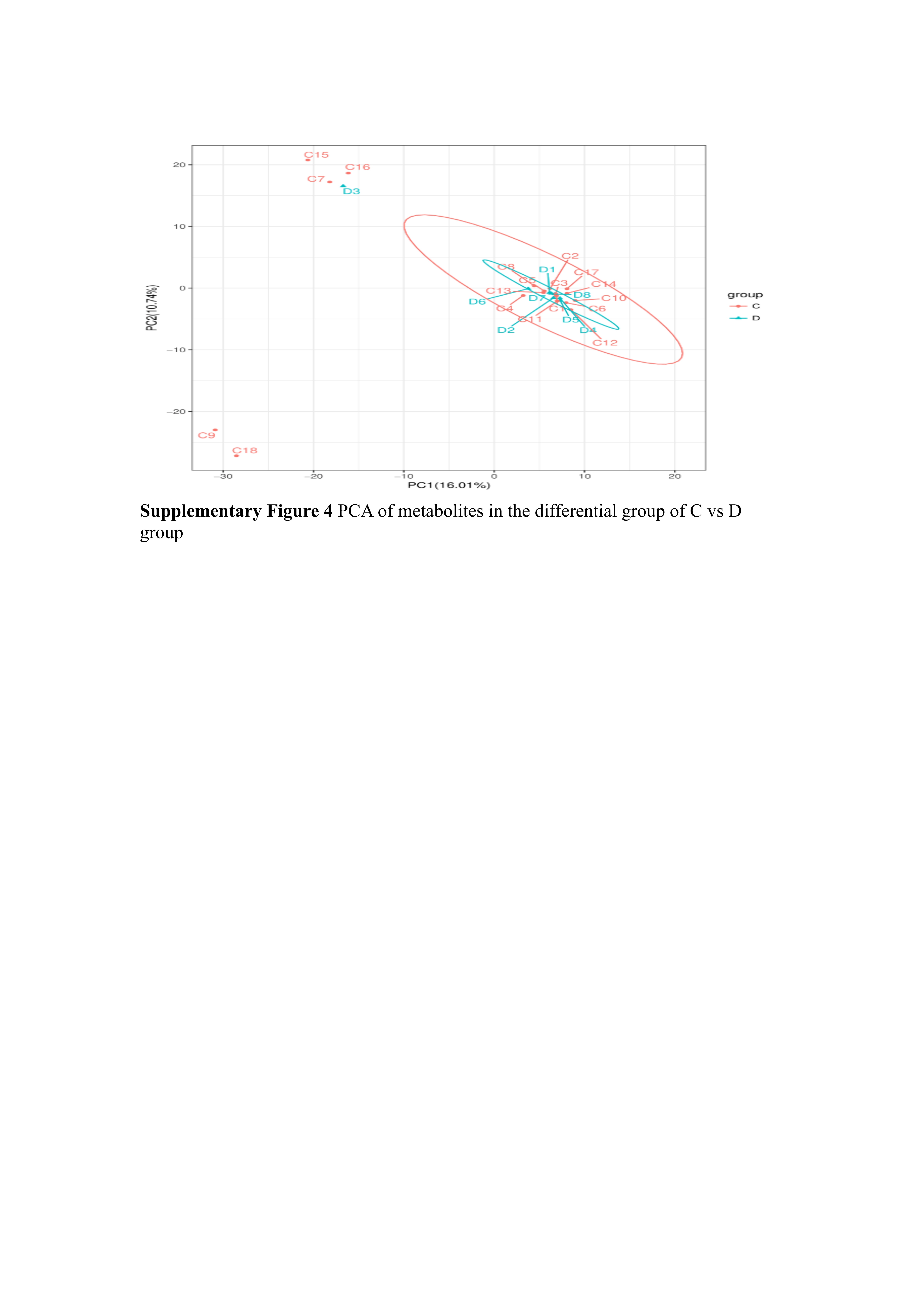

Supplement: Supplementary file 1 [file DataSheet1.zip › Supplementary Figure 4.jpg]

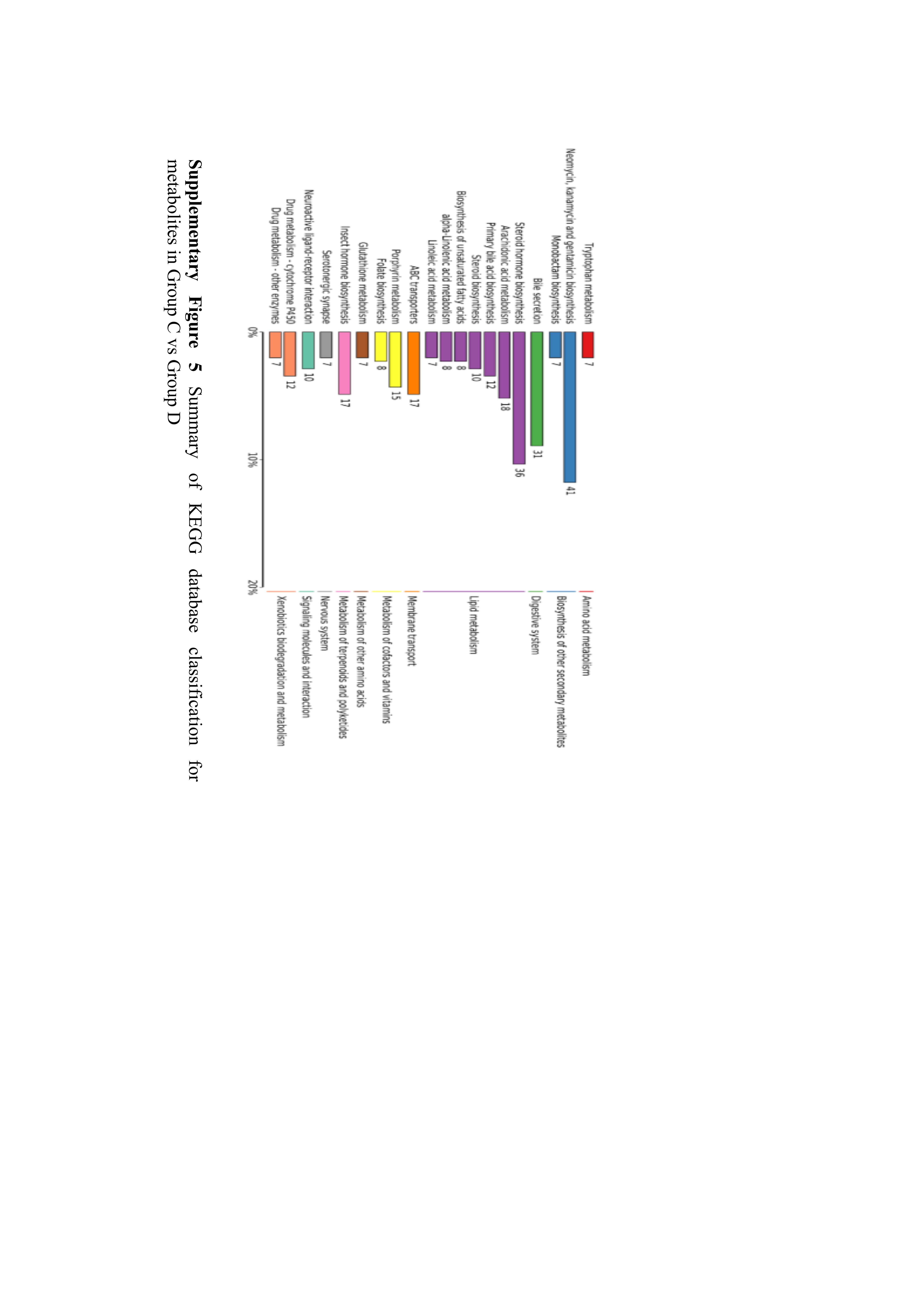

Supplement: Supplementary file 1 [file DataSheet1.zip › Supplementary Figure 5.jpg]

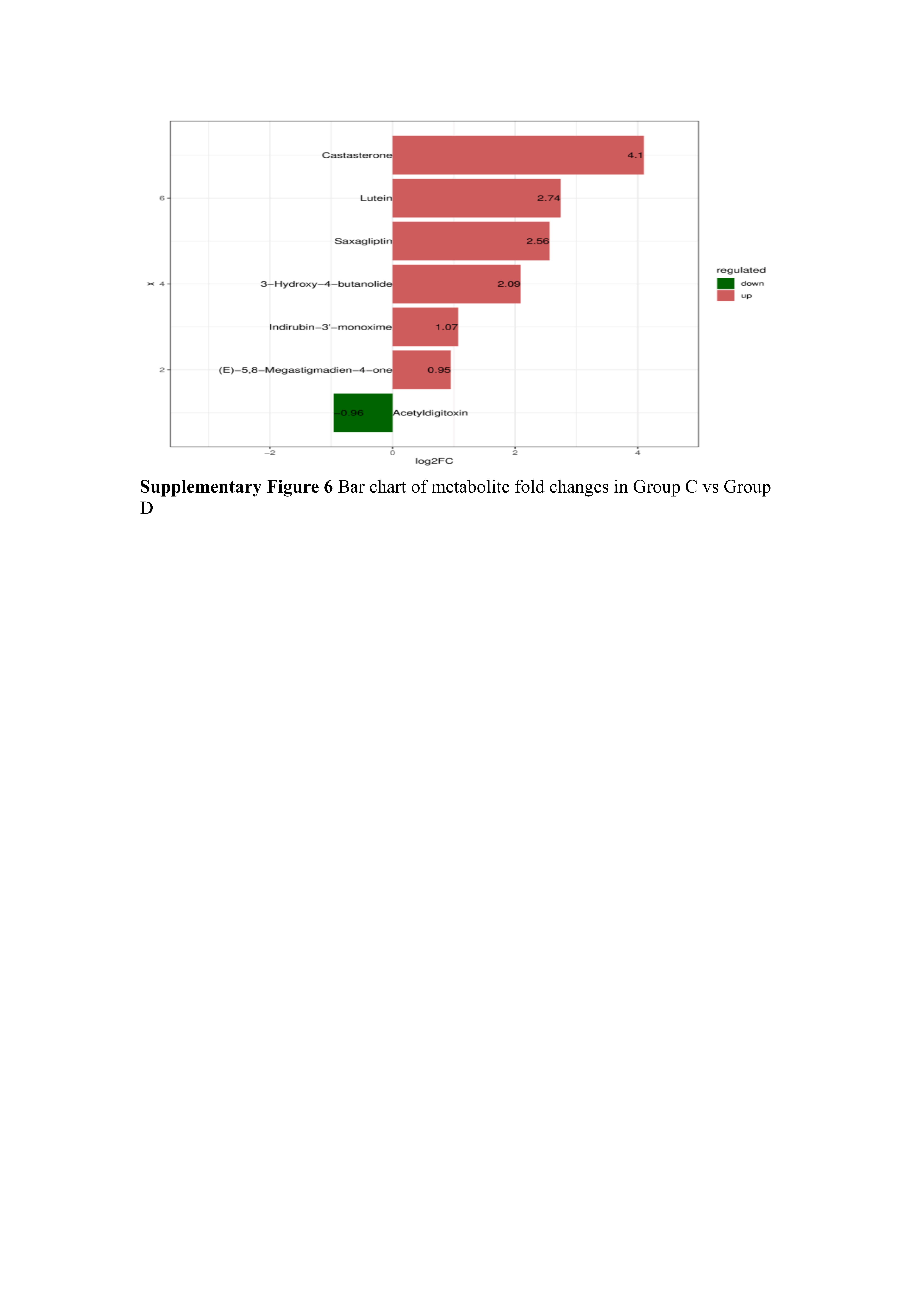

Supplement: Supplementary file 1 [file DataSheet1.zip › Supplementary Figure 6.jpg]

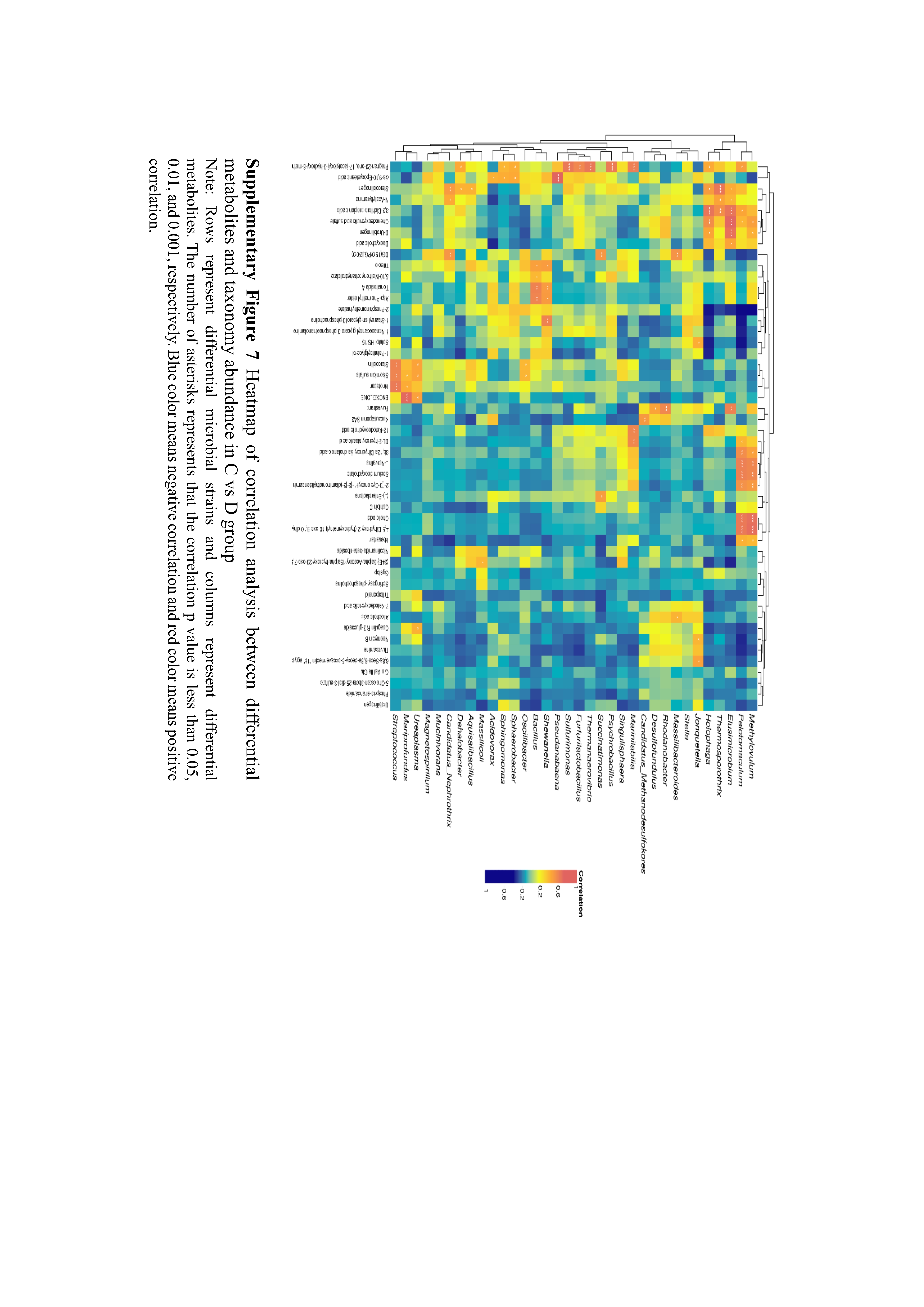

Supplement: Supplementary file 1 [file DataSheet1.zip › Supplementary Figure 7.jpg]

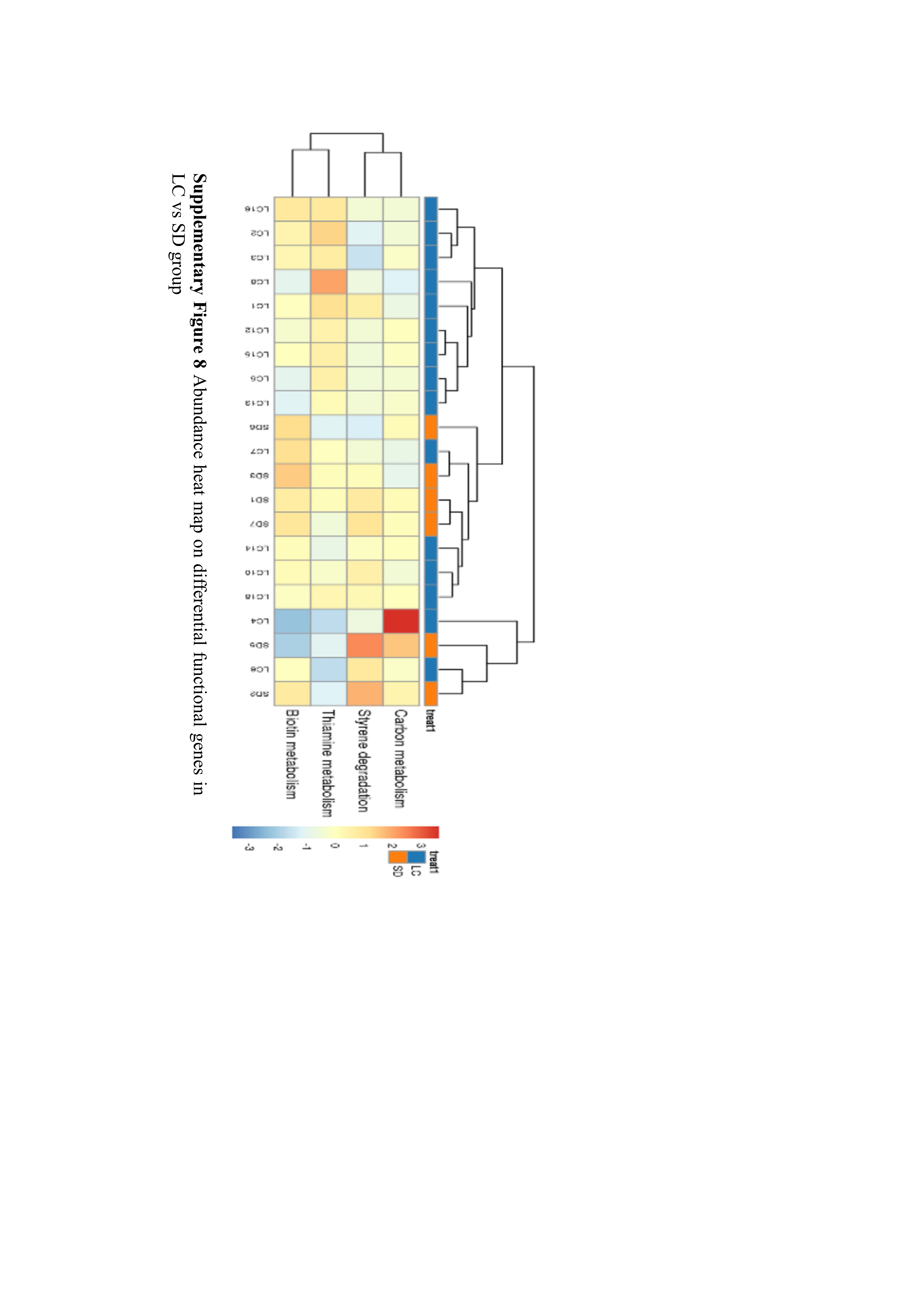

Supplement: Supplementary file 1 [file DataSheet1.zip › Supplementary Figure 8.jpg]

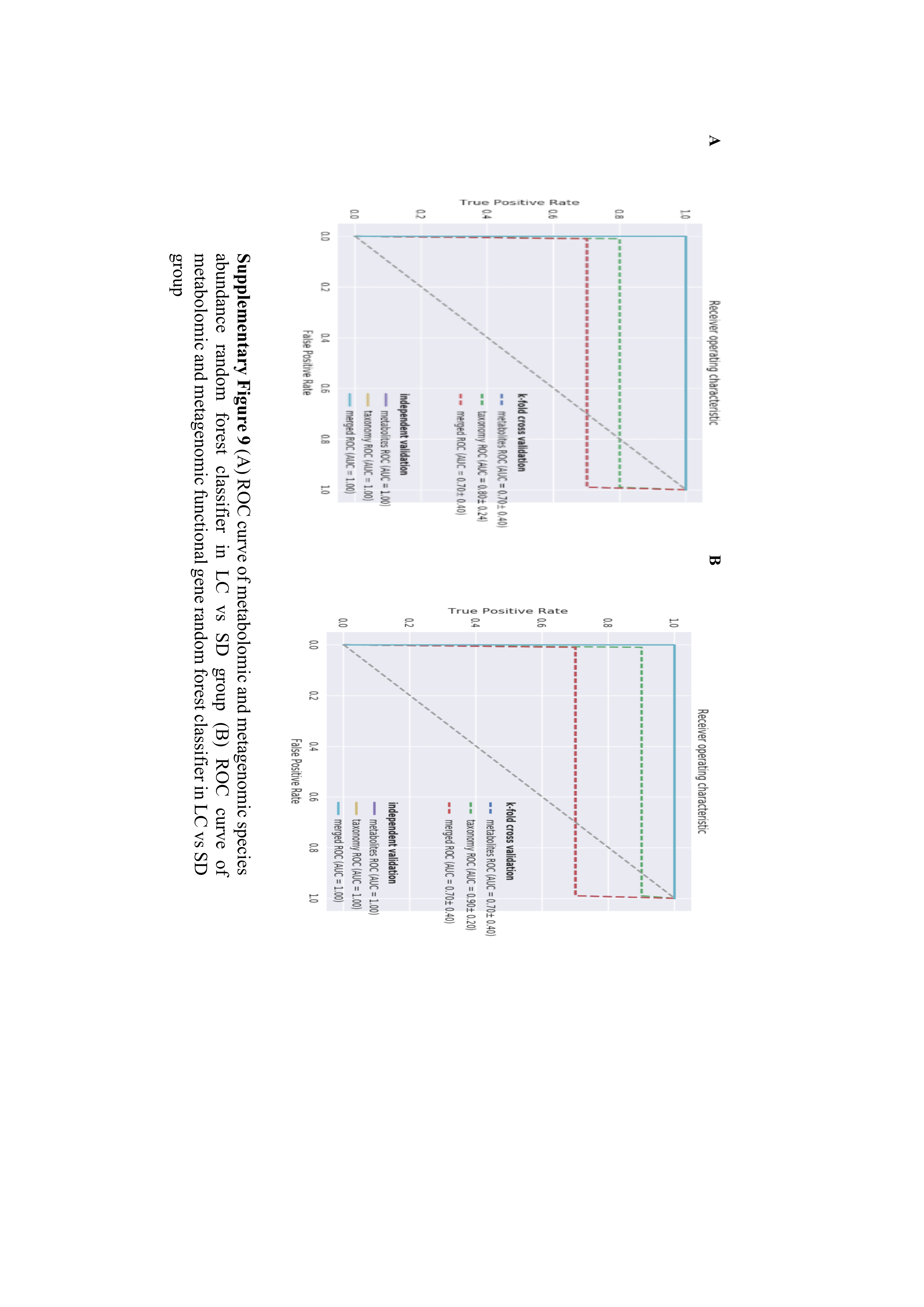

Supplement: Supplementary file 1 [file DataSheet1.zip › Supplementary Figure 9.jpg]

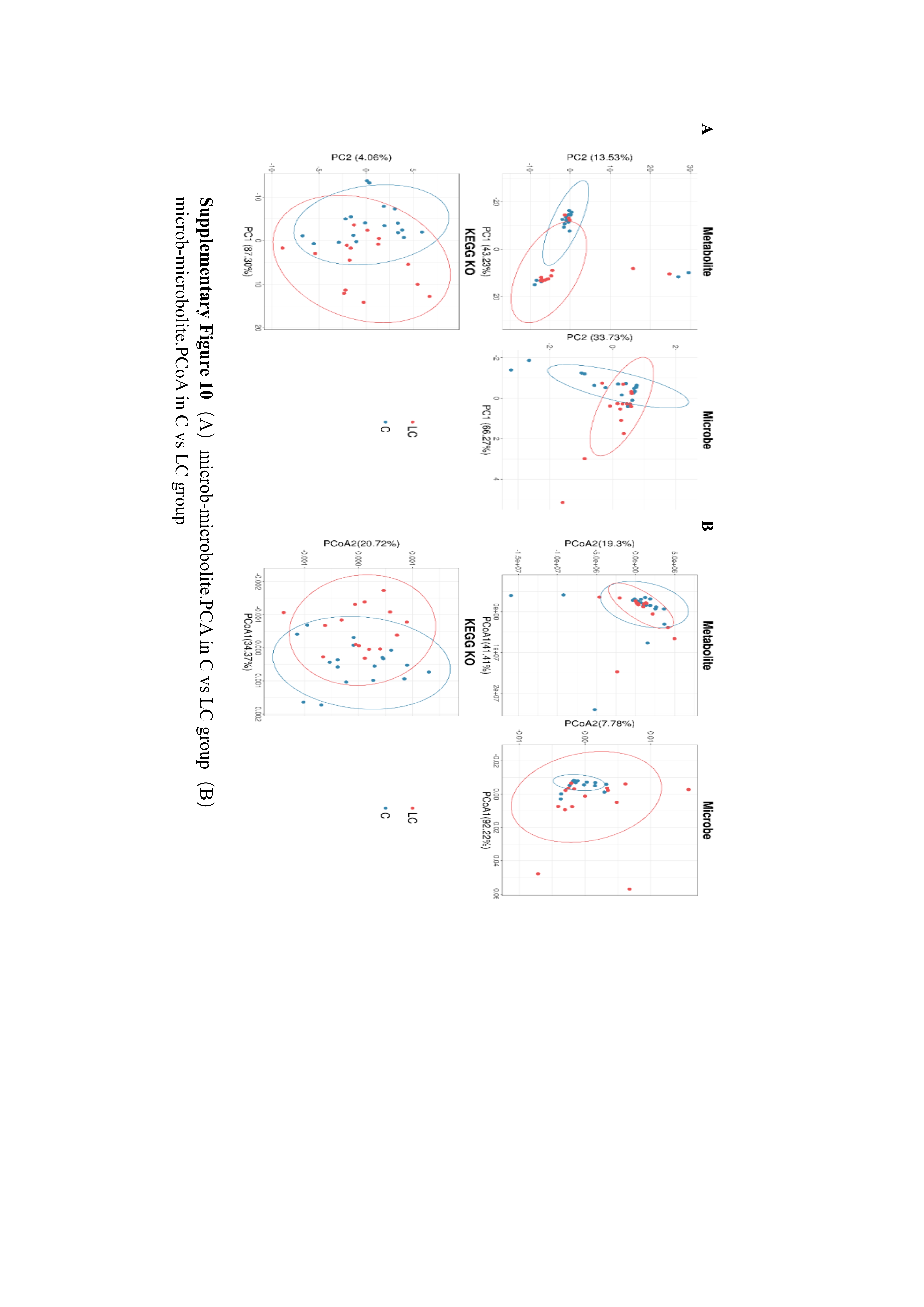

Supplement: Supplementary file 1 [file DataSheet1.zip › Supplementary Figure 10.jpg]

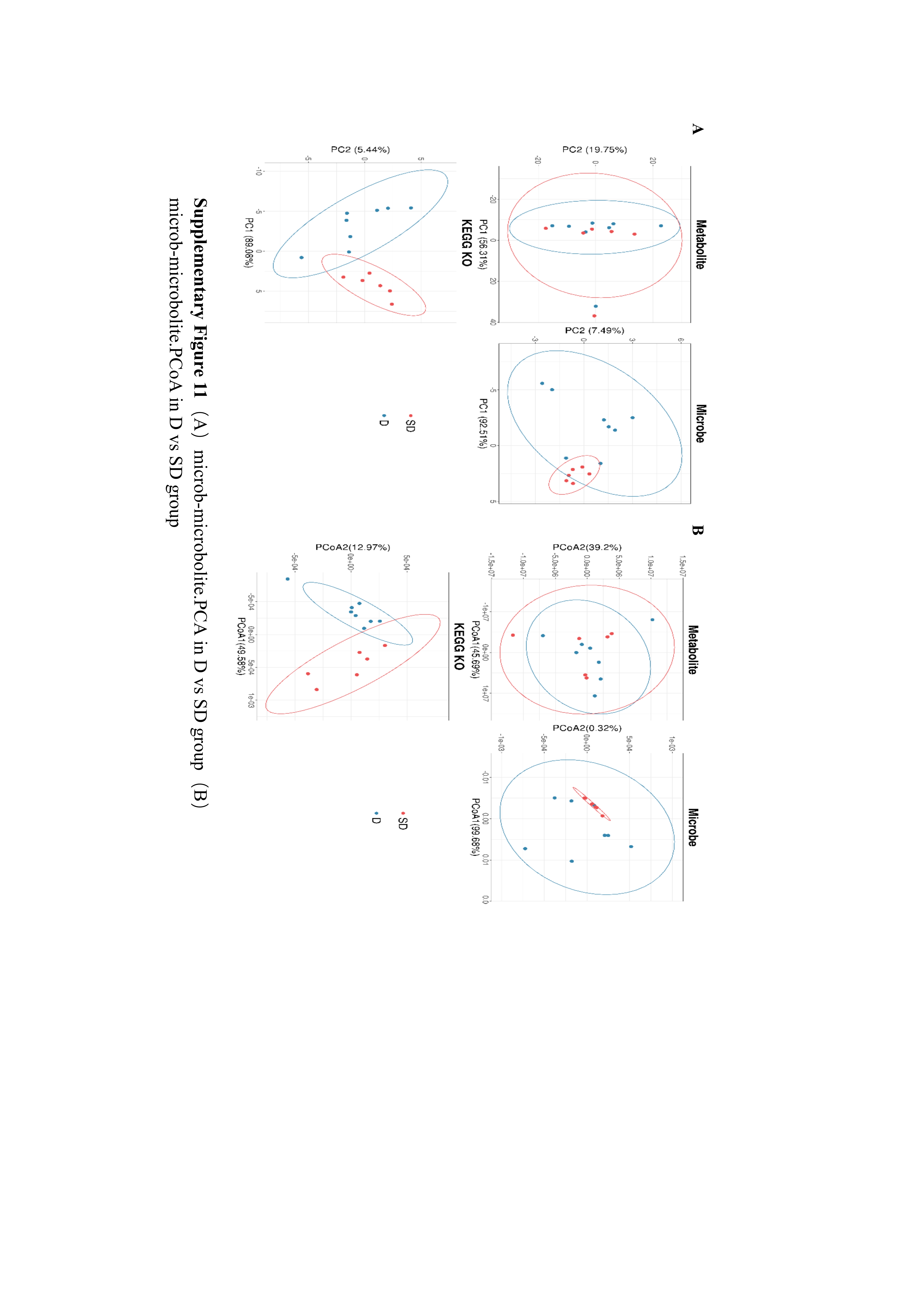

Supplement: Supplementary file 1 [file DataSheet1.zip › Supplementary Figure 11.jpg]

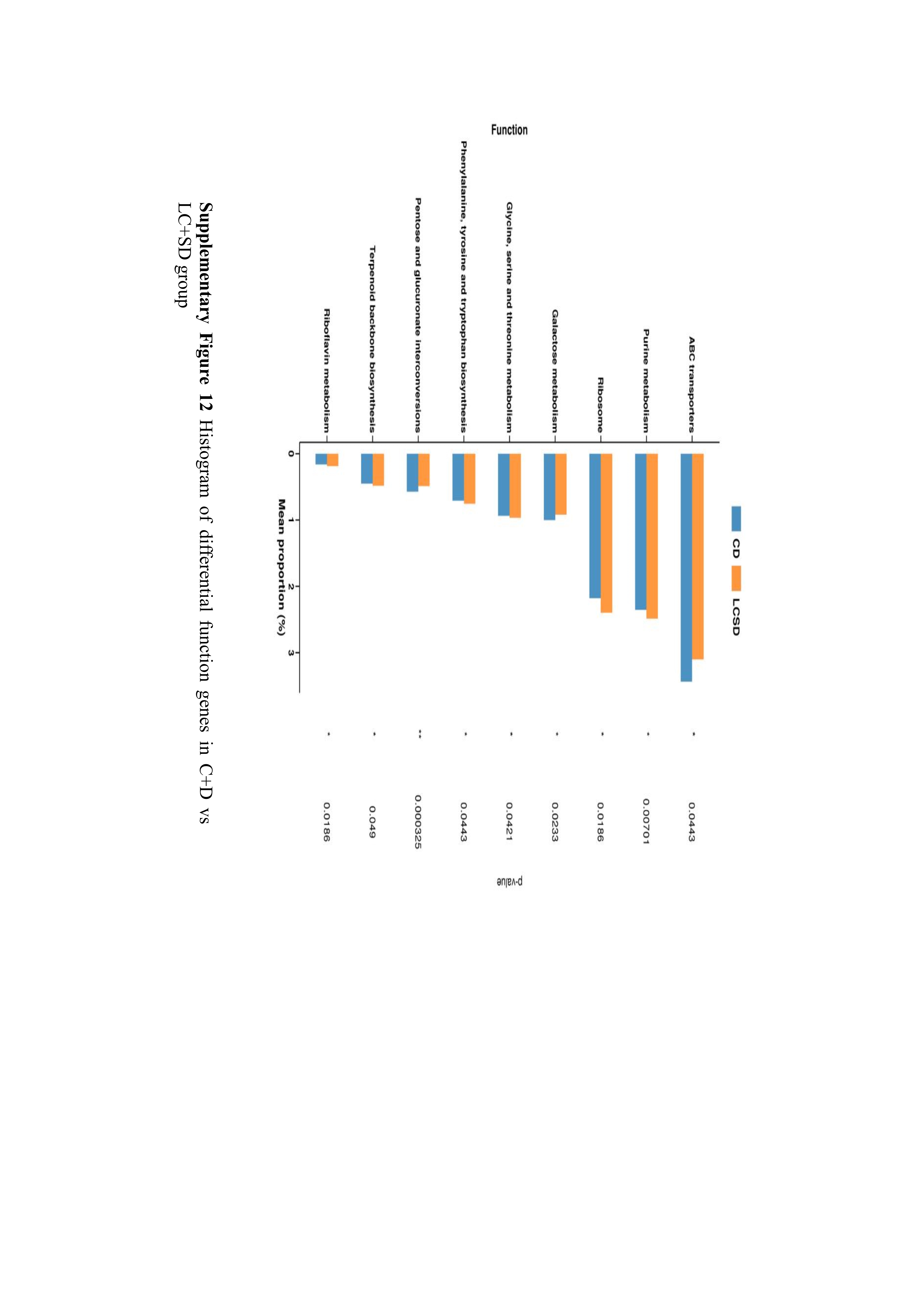

Supplement: Supplementary file 1 [file DataSheet1.zip › Supplementary Figure 12.jpg]

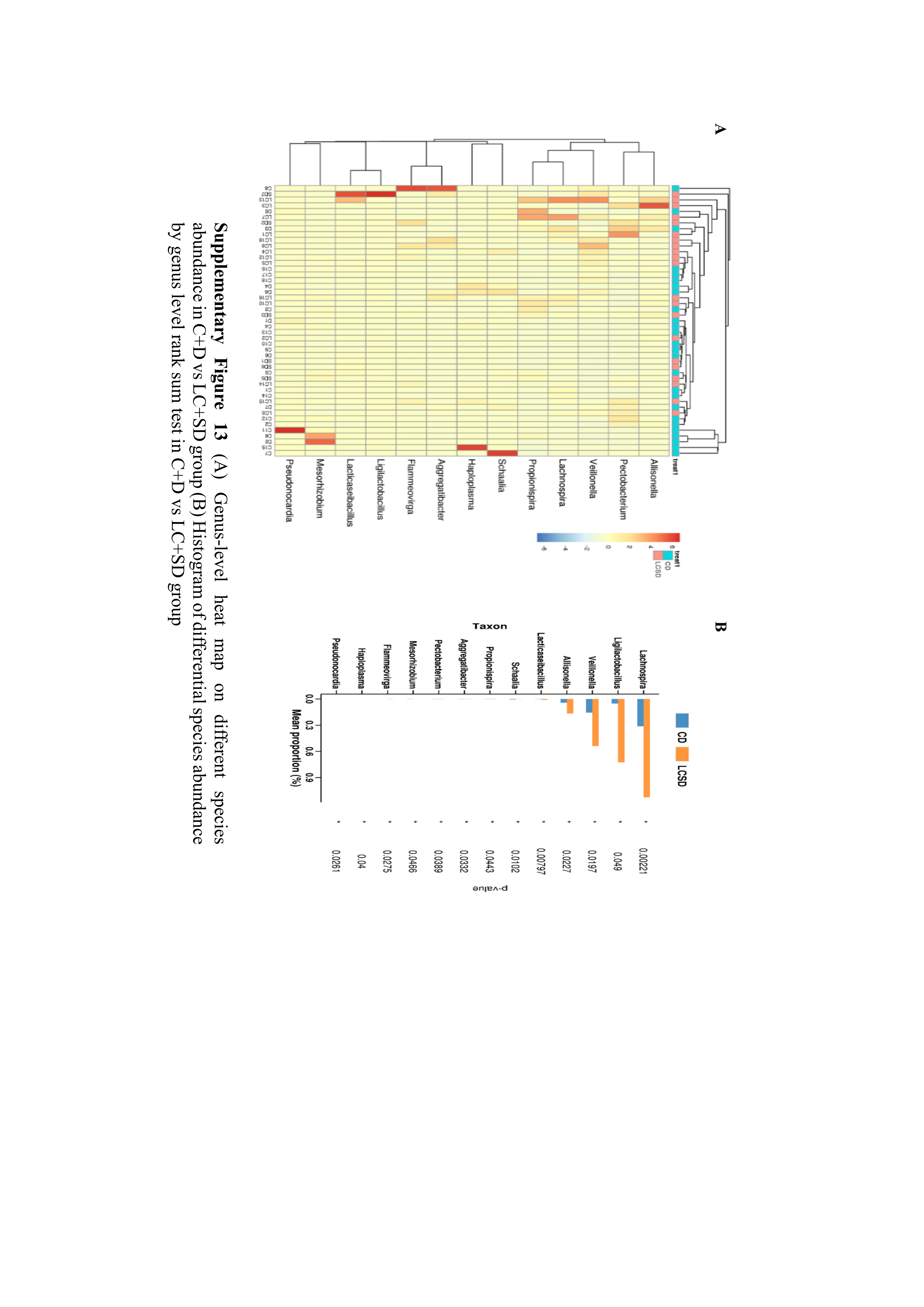

Supplement: Supplementary file 1 [file DataSheet1.zip › Supplementary Figure 13.jpg]

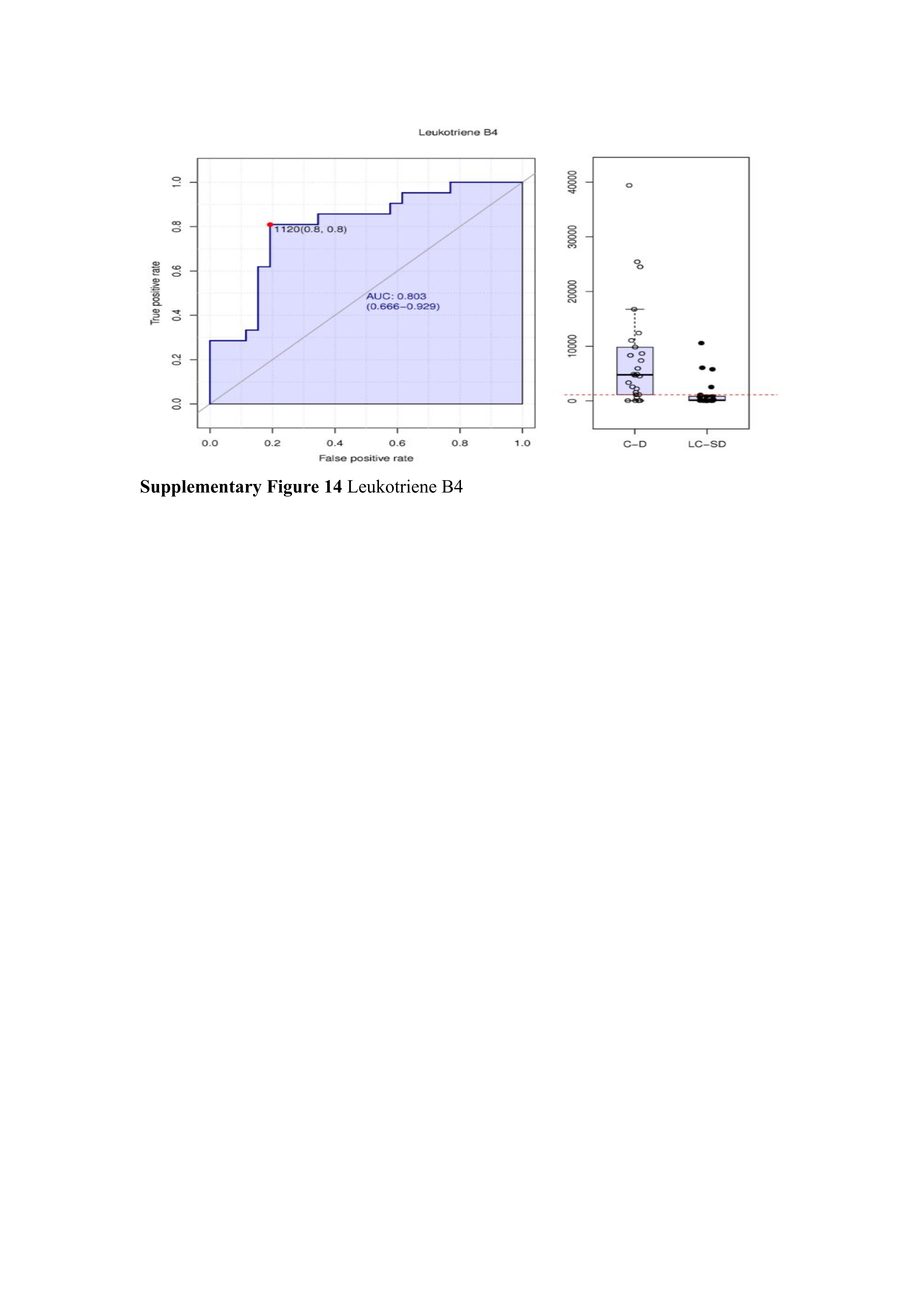

Supplement: Supplementary file 1 [file DataSheet1.zip › Supplementary Figure 14.jpg]
